# Development and validation of MethylCog, a blood DNA methylation proxy for cognition

**DOI:** 10.1002/alz.71421

**Published:** 2026-04-30

**Authors:** Deirdre M. O'Shea, Lily Wang, David Lukacsovich, Devi Dhanekula, Wei Zhang, Conor Galvin, Mahesh Joshi, Lilah Besser, Tatjana Rundek, James E. Galvin

**Affiliations:** ^1^ Comprehensive Center for Brain Health Department of Neurology Miller School of Medicine University of Miami Boca Raton Florida USA; ^2^ Division of Biostatistics Department of Public Health Sciences Miller School of Medicine University of Miami Miami Florida USA; ^3^ Department of Neurology and Evelyn F. McKnight Brain Institute Miller School of Medicine University of Miami Miami Florida USA

**Keywords:** Alzheimer's disease, cognitive ability, DNA methylation, elastic net, epigenetic biomarker, external validation, GrimAge, mild cognitive impairment, MRI, plasma biomarkers

## Abstract

**INTRODUCTION:**

Heterogeneity in cognitive ability increases with age and predicts mild cognitive impairment (MCI) and dementia, but scalable blood‐based biomarkers are lacking. We developed and validated MethylCog, a parsimonious DNA methylation (DNAm) marker of general cognitive ability (g).

**METHODS:**

MethylCog was developed using elastic net regression on principal components analysis (PCA) ‐derived g in a population‐based cohort (*n* = 2,069; training/test split) externally validated (*n* = 112). Criterion validity, MCI discrimination, and specificity relative to GrimAge and Alzheimer's disease (AD) biomarkers were assessed.

**RESULTS:**

MethylCog (29 CpGs) predicted g in the test set (*R* ^2^ = 0.17) and external cohort (*R* ^2^ = 0.13), explaining ∼11% of variance beyond age and sex. MethylCog improved MCI discrimination beyond demographics (ΔAUC = 0.03–0.07) and outperformed GrimAge but did not add value beyond cognitive screeners. Exploratory analyses showed no significant associations with AD plasma biomarkers or MRI measures.

**DISCUSSION:**

MethylCog provides initial evidence that parsimonious DNAm scores can index individual differences in cognitive ability, with potential utility where direct assessment is unavailable.

## Highlights

- MethylCog: A 29‐CpG blood DNA methylation (DNAm) proxy for cognitive ability.
- Replicated in an independent cohort; explained ∼11% of variance beyond age and sex.
- Captured cognition‐specific methylation distinct from GrimAge aging clock.
- No significant associations with Alzheimer's disease (AD) plasma biomarkers or magnetic resonance imaging (MRI) measures.
- Parsimonious score with potential utility in large epidemiologic studies.

## 1 BACKGROUND

Heterogeneity in cognition increases with age and is associated with risk for mild cognitive impairment (MCI), dementia, and mortality in older adults.^1^, ^2^, ^3^, ^4^ General cognitive ability (g), a latent factor reflecting ∼48% of shared variance across diverse cognitive tasks, is a robust life‐course predictor of these outcomes.^5^, ^6^ However, comprehensive neuropsychological assessments, considered the gold standard for measuring g, are time‐intensive and prone to practice effects, limiting their feasibility in large‐scale epidemiological research and retrospective studies where direct testing is unavailable.^7^, ^8^, ^9^ Accessible biological proxies of cognitive ability would complement direct assessment and extend cognitive phenotyping to research contexts where neuropsychological testing is impractical.

DNA methylation (DNAm), an epigenetic mechanism involving the addition of methyl groups to cytosine at CpG dinucleotides, regulates gene expression without altering DNA sequence.^10^, ^11^, ^12^ Stable yet responsive to aging and environmental factors,^13^ DNAm has been increasingly applied in aging research. Blood‐based DNAm aging measures, commonly known as epigenetic clocks, outperform chronological age in predicting disease risk and mortality,^14^, ^15^, ^16^, ^17^ and acceleration of DNAm aging has been associated with life‐course adversity and psychosocial stress.^18^, ^19^ DNAm aging clocks have also shown associations with cognitive outcomes,^20^, ^21^, ^22^ though these tend to be modest, consistent with measures that were not developed to index cognitive variation directly.^14^, ^23^ More targeted approaches using cognition‐specific epigenome‐wide association studies (EWAS) have identified blood‐based CpG sites associated with fluid intelligence, processing speed, memory, and executive function.^24^, ^25^ McCartney et al.^26^ extended this work by developing a genome‐wide DNAm score for g that explained approximately 4% of the variance beyond age and sex in two independent cohorts and showed associations with neuroinflammatory proteins and structural brain magnetic resonance imaging (MRI) measures. Collectively, these findings position DNAm as a compelling candidate biomarker for individual differences in cognitive aging. However, existing DNAm‐based approaches have limited cognitive specificity (i.e., epigenetic aging clocks) or lack the parsimony needed for portability and cost‐effective implementation in large‐scale research (genome‐wide predictors).

To address this gap, we aimed to develop and externally validate a parsimonious blood DNAm proxy of g derived from EWAS‐identified CpGs using penalized regression. We evaluated criterion validity and associations with cognitive diagnosis in both a held‐out test sample and an independent external cohort, characterized its specificity relative to GrimAge, and conducted exploratory analyses examining its relationship to plasma Alzheimer's disease and related dementias (ADRD) biomarkers and structural MRI measures.

## 2 METHODS

### 2.1 Study population

The dataset for model‐development was drawn from the Health and Retirement Study (HRS), a nationally representative longitudinal cohort of U.S. adults aged ≥51 years.^27^ In 2016, a subsample of respondents aged ≥65 years completed the Harmonized Cognitive Assessment Protocol (HCAP), which included an in‐home neuropsychological evaluation and informant interview.^28^ Of 4,425 eligible participants, 3,496 completed the HCAP assessment (79% response rate). Whole‐blood DNAm was assayed on the Illumina EPIC (v1) 850K array as part of the 2016 HRS Venous Blood Study^29^ for 4,018 respondents, of whom 3,966 had complete covariate data.^29^ Of these, 2,462 participants had both HCAP assessment data and DNAm data with complete covariates. After excluding 393 individuals with dementia based on the HCAP diagnostic algorithm,^30^ the final analytic sample included 2,069 participants. All HRS participants provided written informed consent, and study procedures were approved by the University of Michigan Institutional Review Board (IRB).

#### 2.1.1 Healthy Brain Initiative external validation cohort

The external validation sample was drawn from the Healthy Brain Initiative (HBI), an ongoing prospective cohort study of brain aging and dementia risk in community‐dwelling older adults residing in South Florida.^31^ Participants were eligible to enroll in HBI if they had no evidence of dementia (i.e., global Clinical Dementia Rating Scale (CDR) score < 1),^32^ reported either no or only subjective cognitive concerns, had an available study partner, and were able to complete MRI.

**RESEARCH IN CONTEXT**

1. **Systematic review**: We searched PubMed and Google Scholar for studies on blood DNA methylation and cognitive aging and queried the epigenome‐wide association studies (EWAS) Atlas and EWAS Catalog for blood‐based CpG associations with cognitive traits in adults. DNA methylation (DNAm) aging clocks show modest cognitive associations but were not developed to index cognition directly. Prior cognition‐focused EWAS identified relevant loci, and one genome‐wide score explained ∼4% of external cognitive variance.
2. **Interpretation**: We developed and externally validated MethylCog, a 29‐CpG blood DNAm marker of general cognitive ability derived from prior cognition‐related EWAS loci. MethylCog explained between 13%−17% of cognitive variance across cohorts and showed greater cognitive specificity than GrimAge.
3. **Future directions**: Future work should test whether MethylCog predicts longitudinal cognitive decline, replicates in larger and more diverse cohorts, and remains distinct from AD plasma and neuroimaging biomarkers across the full clinical spectrum.

DNAm data were available for a subset (*n* = 112) of HBI participants with full cognitive data. Participants were excluded if they lacked consent for data/specimen storage, were unable to identify a study partner, or had a diagnosis of dementia. Research diagnoses of cognitively normal or MCI are assigned based on clinical assessments, medical history, and CDR scores, with final determinations reached in monthly consensus conferences. In the final analytic sample, no participants had a diagnosis of dementia. All participants provided written informed consent, and study procedures were approved by the University of Miami IRB.

### 2.2 Cognitive assessments

#### 2.2.1 HRS‐HCAP

Participants completed a comprehensive, in‐home neuropsychological battery covering episodic memory, visuoconstruction, attention/processing speed, executive function, nonverbal reasoning, and semantic fluency. Brief task descriptions are provided in Table S1 with full administration and scoring details available in prior HCAP publications and original test manuals.^28^, ^30^ All test scores were standardized and entered into a principal components analysis (PCA) to derive a general cognitive ability factor (g‐factor), as described in the Statistical Analyses section.

#### 2.2.2 HBI

Cognitive testing for HBI followed a standardized in‐person neuropsychological protocol,^31^ using the Uniform Data Set (UDS 3.0) from the National Institute on Aging Alzheimer's Disease Research Center program.^33^ As in HRS‐HCAP, standardized test scores were analyzed using the same PCA framework to derive a g‐factor for external validation (see the Statistical Analyses section). Brief task descriptions are provided in Table S2.

### 2.3 DNAm assays and preprocessing

#### 2.3.1 HRS‐HCAP

DNAm data for the HRS cohort were obtained from the National Institute on Aging Genetics of Alzheimer's Disease Data Storage Site (NIAGADS).^34^ The original experiment utilized 500 ng of genomic DNA, which was assayed using the Illumina Infinium Methylation EPIC Bead Chip (v1.0)^35^ at the University of Minnesota Genomics Center. Raw IDAT files were reprocessed to ensure harmonization with the HBI dataset. Preprocessing was conducted in R using standard pipelines. Probes were removed if they were previously identified as cross‐reactive,^36^ did not have a “cg” identifier, or contained a single nucleotide polymorphism (SNP) with a minor allele frequency ≥ 0.01 within 5 bp of the probe (implemented with the “DMRcate” function “rmSNPand CH”, “parameters” dist = 5, mafcut = 0.01″).^37^ Missing values and failed probes (detection *p*‐values > 0.01) were imputed using the methyLImp2 R package;^38^ designed specifically for methylation arrays. At the sample level, QC included selection of samples with good bisulfite conversion efficiency (> 85%) and verification that predicted sex (using the wateRmelon package;^39^ matched reported sex.

Normalization was performed using β‐mixture quantile normalization (BMIQ) method^40^ implemented in watermelon R package. To reduce technical confounding, batch effects were corrected using the “Harman” R package.^41^ PCA was then conducted on the sample‐by‐CpG matrix (using methylation *M*‐values). For each sample we computed *Z*‐scores on the first two principal components (PC1 and PC2). A sample was flagged as an outlier if its *Z*‐score exceeded ± 3 standard deviations on either PC1 or PC2 and was subsequently removed. Finally, immune cell proportions were estimated using the “EpiDISH” R package.^42^

#### 2.3.2 HBI

The HBI DNAm data consisted of samples from two batches. Genomic DNA (500 ng) was assayed on the Illumina Infinium MethylationEPIC v2.0 BeadChip^43^ at the Center for Genomic Technology, University of Miami. DNA was bisulfite‐converted using the EZ‐96 DNA Methylation Kit (Zymo Research, Irvine, CA). DNAm preprocessing and quality control for the HBI dataset followed the same general pipeline described for the HRS‐HCAP discovery set to ensure methodological harmonization across cohorts. For the EPIC v2.0 data, probes not present in the EPIC v2 annotation or not beginning with the standard “cg” prefix (e.g., non‐CpG probe types such as nv nucleotide‐variant probes) were excluded during preprocessing. When multiple probes targeted the same CpG locus, values were averaged after imputation and before normalization. The two HBI datasets were merged and preprocessed together and batch corrected. All QC parameters (e.g., detection *p* value > 0.01, bisulfite conversion efficiency > 85%) were identical to those used in HRS‐HCAP to ensure harmonization.

### 2.4 Candidate CpG selection

To restrict model development to CpG sites previously linked to normative cognitive variation, candidate CpGs were identified from published epigenome‐wide association studies indexed in the EWAS Atlas^44^ and EWAS Catalog.^45^ Search terms included “cognition,” “cognitive ability,” “fluid intelligence,” “cognitive domains,” “memory,” “executive function,” “processing speed,” and “attention.” Studies restricted to Alzheimer's disease, MCI, or other neurodegenerative diagnoses were excluded to avoid capturing disease‐specific epigenetic signatures. CpGs were further restricted to probes identified from blood samples in adult or older adults, present on the Illumina 450K array for comparability with existing epigenetic studies^46^ and those that passed all quality control filters in both the HRS‐HCAP and HBI datasets. After applying these criteria, 558 CpGs remained for model development. The full list of CpGs, including genomic annotation and source references, is presented in Table S3.

### 2.5 Comparator DNAm measure: GrimAge

DNAmGrimAge (GrimAge), a widely used epigenetic clock predictive of all‐cause mortality,^15^ was used as a comparator to evaluate the specificity of the DNAm‐based measure of g, herein named “MethylCog”. GrimAge was selected a priori as the epigenetic clock comparator to provide a parsimonious benchmark against an established DNAm aging measure.^22^ In prior HRS‐HCAP analyses, it was the only one of four DNAm clocks associated with cognitive outcomes,^47^ and subsequent studies in the same dataset further supported its associations with memory and other cognitive measures.^20^, ^21^

In the HRS, GrimAge values were obtained from the HRS restricted data release, which was computed using the original GrimAge algorithm based on DNAm‐derived surrogates of smoking pack‐years and multiple age‐related plasma proteins.^48^

In the HBI, GrimAge was recalculated using a principal component (PC)‐based reexpression to improve cross‐platform portability between the Illumina EPIC v1.0 (HRS) and EPIC v2.0 (HBI) arrays. Following the PC‐clock framework,^49^ β‐value matrices were projected onto reference eigenvectors corresponding to the GrimAge probe set, and the resulting PCs were combined to generate GrimAge estimates using the methylclock R package [version 0.7.5; ^50^].

### 2.6 Blood‐based biomarkers of ADRD and apolipoprotein E genotyping (HBI cohort)

Venous blood was collected at the same visit as cognitive testing. All specimens were processed according to HBI standard operating procedures and stored at −80°C until analysis.

Blood plasma amyloid‐ and tau‐related biomarkers were quantified by C2N Diagnostics (St. Louis, MO, USA) using a mass spectrometry‐based platform.^51^ This panel included amyloid‐β 42 (Aβ42), amyloid‐β 40 (Aβ40), phosphorylated tau at threonine 181 (pTau181), phosphorylated tau at threonine 217 (pTau217), and the pTau217/npTau217 ratio. The Aβ42/40 ratio was derived as an index of amyloid burden, with lower values indicating a greater likelihood of amyloid pathology. Mass spectrometry by C2N was performed to estimate proteotyping to determine apolipoprotein E (APOE) ε2/ε3/ε4 carrier status; APOE ε4 carrier status (any ε4 vs none) was used in the present analyses. Assays were conducted in batches with internal controls, and all runs met manufacturer‐recommended quality control criteria, including acceptable intra‐ and inter‐assay coefficients of variation. Laboratory personnel were blinded to all clinical and cognitive data.

Additional neurodegeneration‐ and inflammation‐related biomarkers were quantified in the HBI laboratory using the Quanterix Simoa™ SR‐X platform^52^ with commercially available assay kits (single molecule array [Simoa] assay kits from Quanterix Corp). Glial fibrillary acidic protein (GFAP) was measured as a marker of astroglial activation, and neurofilament light chain (NfL) as a marker of axonal injury.^53^ Concentrations were expressed in picograms per milliliter (pg/mL).

### 2.7 Neuroimaging assessment (HBI)

Participants underwent structural brain MRI on a GE 3T 750 W scanner. The protocol included a high‐resolution 3D sagittal magnetization prepared rapid gradient echo (MPRAGE) sequence with axial and coronal reconstructions to quantify regional brain volumes, as well as axial 3D T2 fluid‐attenuated inversion recovery (FLAIR) and 2D T2* sequences for white matter hyperintensity (WMH) and microhemorrhage assessment. Quantitative volumetric measures were derived using the United States Food and Drug Administration (FDA) ‐cleared Combinostics® cNeuro Suite, which provides automated segmentation of cortical and subcortical structures and WMH. The AI‐driven Combinostics cMRI suite performs comparably to manual segmentation (Pearson *r* = 0.84–0.99).^54^ For the present study, total gray matter (GM) volume, total white matter (WM) volume, bilateral hippocampal volume, and periventricular WMH (pWMH) volume were obtained as the primary MRI measures. Total intracranial volume (ICV) was estimated by the Combinostics pipeline, and all volumetric measures (GM, WM, hippocampus, pWMH) were normalized for head size by dividing each individual volume by that participant's ICV and multiplying the result by the sample mean ICV. Periventricular WMH were defined as WMH lesions adjacent to the lateral ventricles on FLAIR images. We focused on pWMH rather than total WMH volume because WMH burden in the periventricular region is considered a more specific marker of small vessel–related WM damage and has been more consistently linked to cognitive decline in prior work.^55^, ^56^

### 2.8 Statistical analysis

**FIGURE 1:**
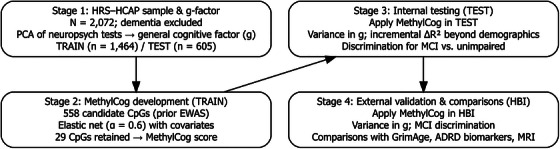
Analytic workflow for MethylCog development, internal testing, and external validation across HRS‐HCAP and HBI cohorts. HCAP, Harmonized Cognitive Assessment Protocol; HRS, Health and Retirement Study.

As shown in Figure 1, analyses proceeded in four stages: (1) PCA of neuropsychological tests to derive measured g; (2) elastic net—based development of the MethylCog score in the HRS‐HCAP training set; (3) internal testing in a held‐out HRS‐HCAP test set; and (4) external validation and comparison with epigenetic aging measures and ADRD biomarkers in the HBI cohort.

#### 2.8.1 Descriptives statistics

For each dataset, we summarized demographic, clinical, cognitive, and DNAm characteristics using means and standard deviations for continuous variables and frequencies and percentages for categorical variables. To evaluate representativeness of the analytic subsets, we compared the HRS‐HCAP test set with the full HRS sample at the 2016 wave (same year as HCAP) aged 65 years and older and compared the HBI validation subset with the full eligible HBI cohort (*n* = 543). Continuous variables were compared using Welch two‐sample *t*‐tests, and categorical variables were compared using chi‐squared tests or Fisher's exact tests when cell counts were small.

#### 2.8.2 Data preparation and sample splitting (HRS‐HCAP)

The HRS‐HCAP dataset was split (70/30) into a training set (*N* = 1,464) and a test set (*N* = 605) using stratified sampling to maintain balance across cognitive diagnosis, ethnicity, sex, and education. Distributions of age, sex, race/ethnicity, education, and MCI status were comparable across the training and test set (Table 1).

**TABLE 1:** Sample characteristics.

Parameter		Training set(*n* = 1, 464)	Test set(*n* = 605)	Training versus test *p* value	HBI(*N* = 112)
Age	Mean (SD),	75.22 (7.01),	75.20 (7.08),	0.953	68.37 (10.44),
	Range	65.00–98.00	65.00–96.00		46.00–89.00
Education	Mean (SD),	12.80 (3.11),	12.89 (3.02),	0.541	16.21 (2.10),
	Range	0.00–17.00	0.00–17.00		11.00–20.00
g	Mean (SD),	0.00 (1.00), ‐	0.00 (1.00),	—	−0.00 (1.00),
	Range	4.40–2.15	−3.01–2.03		−3.04–−1.98
Female	*n* (%)	828 (56.6%)	345 (57.0%)	0.845	78 (69.6%)
Race				0.011	
White	*n* (%)	1165 (79.6%)	513 (84.8%)		96 (85.7%)
Black/AA	*n* (%)	214 (14.6%)	75 (12.4%)		—
Other race	*n* (%)	79 (5.4%)	17 (2.8%)		16 (14.3%)
Hispanic	*n* (%)	156 (10.7%)	58 (9.6%)	0.468	10 (8.9%)
MCI	*n* (%)	348 (23.8%)	141 (23.3%)	0.821	37 (33.0%)
APOE ε4	*n* (%)	318 (23.3%)	131 (23%)	0.881	32 (29%)

*Note*: HBI = Healthy Brain Initiative. Age and education are reported in years as mean (SD) and range. g = standardized composite general cognitive ability score (higher scores indicate better cognitive performance). Sex, race, Hispanic ethnicity, and MCI status are reported as numbers (percentages). “Other race” includes participants who self‐identified with racial categories other than White or Black/African American (e.g., Asian, Native American, Pacific Islander, multiracial, or other). Percentages are calculated within each sample and variable based on non‐missing data. *p* values reflect training‐versus‐test comparisons only. Continuous variables were compared using Welch two‐sample t‐tests, and categorical variables were compared using chi‐squared tests. For race, the *p* value reflects the overall three‐category race comparison.

Abbreviations: AA, African American; APOE, apolipoprotein E; HBI, Healthy Brain Initiative; MCI, mild cognitive impairment; SD, standard deviation.

#### 2.8.3 Derivation of the g

A PCA was conducted on z‐standardized neuropsychological test scores for HRS‐HCAP (prior to data splitting) and HBI to derive the measure of g. The first unrotated principal component (PC1) was extracted as the measure of g

#### 2.8.4 DNAm model development (elastic net; the training set)

An elastic net regression model was employed to develop MethylCog from whole blood CpG sites in the HRS training set. The dependent variable was the standardized measured g. All CpGs (from predefined set of 570) that were also present in both the training data and the independent HBI cohort were eligible predictors; this resulted in a final candidate set of 558 CpGs (CpGs present in the training set but not in HBI were excluded to ensure transportability of the final model and were cataloged separately). In the training set, CpG beta values were standardized using probe‐specific means and standard deviations estimated in the training set.

#### 2.8.5 Model specification and tuning

Cell‐type proportions (granulocytes [GR]]), natural killer cells [NK], B cells [B], CD4 T cells [CD4], monocytes [MO]; CD8 T cells [CD8] as the reference), sex, and batch (numeric plate identifier) were included as unpenalized covariates. Cell fractions and batch indicators were z‐standardized (cells) or dummy‐coded with one reference category dropped (sex and batch), respectively. Although the DNAm data underwent initial batch correction using *Harman* during preprocessing, we included the plate‐coded batch as an unpenalized covariate in the elastic‐net model to mitigate any residual technical variance that may have persisted and ensure the selection of CpGs was driven by biological signal. To reduce the influence of poor‐quality methylation profiles, we removed training set participants whose proportion of missing CpG values exceeded 10% across the shared CpG pool. This filter did not result in the exclusion of any participants, confirming the high quality of the preprocessed training data. Since analyses were restricted to 450K array probes for cross‐study compatibility while samples were assayed on EPIC arrays, unmeasured 450K probes were set to zero after standardization. Elastic net models were fit using the glmnet package^57^ with a Gaussian family.

For each candidate model, a 10‐fold cross‐validation with a fixed, randomly generated fold assignment (seed = 20251014) was used to select tuning parameters. A joint search over the mixing parameter alpha (α ) and the penalty parameter λ (lambda) was conducted by fitting separate cv.glmnet models for α values on a 0–1 grid (increments of 0.1) and using the 1‐SE rule within each fit to identify the corresponding $\lambda_{1SE}$. The optimal α‐λ pair was defined as the combination that minimized cross‐validated mean squared error (CV_MSE). The final model selected a chosen α of 0.6 and a λ of 0.082, yielding a parsimonious set of 29 CpGs.

#### 2.8.6 Scoring and reproducibility

The final elastic net model was then refit using the full training set design matrix at the chosen α and λ values, with all CpG predictors penalized (penalty factor = 1) and all covariates unpenalized (penalty factor = 0). From this final model, the intercept and all regression coefficients were extracted and saved separately from all nonzero coefficients for CpG probes which were then used as the weighting scheme to compute MethylCog across datasets. The final set of selected CpGs and their corresponding weights are shown in Figure S1 and Table S4

For each dataset (i.e., training, test, HBI), CpG matrices were aligned to the training set CpG pool, standardized using the training set means and standard deviations, and restricted to participants with ≤10% missing CpG values in this pool. MethylCog scores were computed as the linear combination of standardized CpG values and their corresponding beta weights (covariate effects were not applied outside the training set), with any remaining nonfinite entries set to zero before scoring. Raw scores were then standardized to the training set distribution by subtracting the training set mean of the CpG only score and dividing by the training set standard deviation, producing zscaled MethylCog values that were directly comparable across datasets. The MethylCog score was evaluated against the measured g using *R* ^2^, root mean squared error (RMSE), mean absolute error (MAE), Pearson's r, and Spearman's ρ. Model artifacts (CpG weights, scaling parameters, fold assignments, and run metadata) and scored datasets were saved to support reproducibility.

#### 2.8.7 Group differences in MethylCog

Group differences in MethylCog scores were examined separately in the test set and HBI set using Welch's two‐sample *t*‐tests for binary variables, Welch's analysis of variance (ANOVA) for categorical variables with three or more levels, and Pearson's product‐moment correlations for continuous variables.^58^ All analyses used an alpha level of 0.05. Box plots for visualizing were also generated.

#### 2.8.8 Criterion validity for continuous cognition

To quantify criterion validity, we examined the extent to which MethylCog explained variance in g beyond demographic factors in test and HBI sets. In each dataset, we fit a base linear regression model including age, sex, and education, followed by a model that added MethylCog. Incremental validity was assessed using Δ*R* ^2^, semi‐partial *R* ^2^ for MethylCog, and corresponding regression coefficients with 95% confidence intervals (CIs).

#### 2.8.9 Discrimination for cognitive diagnosis (cognitively unimpaired vs. MCI)

To evaluate the clinical potential of MethylCog for discriminating cognitive status, we modeled diagnosis (0 = cognitively unimpaired, 1 = MCI) in the test and HBI sets using logistic regression. We compared a demographics‐only base model (age, sex, education) to a model including demographics and MethylCog. For incremental validity beyond cognitive screeners, three models were conducted: (1) base model; (2) base model and the cognitive screener (Mini‐Mental State Examination [MMSE] in training/test sets, Montreal Cognitive Assessment [MoCA] in HBI); and (3) base model, cognitive screener, and MethylCog. Odds ratios (ORs) with 95% CIs for MethylCog, area under the receiver operating characteristics curve (AUC), and changes in AUC tested using DeLong's method,^59^ as well as likelihood‐ratio tests (Δχ^2^) comparing nested models were estimated.

#### 2.8.10 Sensitivity analyses

To evaluate the robustness of the MethylCog score, we performed a sensitivity analysis in the test and HBI sets by excluding extreme outliers defined by a dual‐criterion Z‐score ≥ 3 or MAD‐Z (Median Absolute Deviation Z‐score, a robust measure of deviation less influenced by extreme values) ≥ 3.5.^60^ To assess effect modification sensitivity analyses adjusting for APOE ε4 carrier status, sex and race/ethnicity and tested corresponding interaction terms. In addition, exploratory mediation analyses examined whether MethylCog statistically mediated associations between race/ethnicity and cognitive outcomes using nonparametric bootstrapping (1,000 iterations).

#### 2.8.11 Assessment of independence from epigenetic aging clocks

To evaluate whether MethylCog captured variance distinct from general epigenetic aging, MethylCog was compared with the GrimAge clock. Within each cohort, GrimAge and MethylCog were residualized on chronological age to obtain the GrimAgeResid and MethylCogResid. Higher GrimAgeResid reflects accelerated epigenetic aging; higher MethylCogResid reflects better‐than‐expected DNAm‐derived general cognitive ability for age. Correlations between the residualized metrics were examined using bivariate Persons correlations. Hierarchical linear regression models were then fitted to assess incremental variance (Δ*R* ^2^) in g explained by MethylCogResid beyond the base model with GrimAgeResid. To examine the discriminative ability of MethylCogResid in cognitive impairment, beyond a base model with the GrimAgeResid, a binary logistic regression model was conducted (cognitive status as the outcome), with AUC and ΔAUC used to assess incremental discrimination.

#### 2.8.12 Associations with ADRD blood biomarkers and MRI (HBI only)

In the HBI, exploratory analyses were conducted to assess associations between MethylCog and g with ADRD blood biomarkers and MRI‐derived measures. Prior to analysis, right‐skewed variables, including GFAP, NfL, pTau181, the pTau217/npTau217 ratio, and periventricular WMH volume, were natural logarithm‐transformed to approximate normality. The Aβ42/40 ratio was analyzed on its untransformed scale given no evidence of skew. Pairwise partial correlations, adjusted for age and sex, were computed between each predictor (MethylCog, g) and (a) plasma biomarkers (Aβ42/40 ratio, GFAP, NfL, pTau181, and pTau217/npTau217 ratio) and (b) intracranial volume‐normalized MRI measures (total GM volume, total WM volume, total hippocampal volume, and pWMH volume). Missing data were addressed using pairwise‐complete observations, with each association reported as the partial correlation coefficient (r), *p* value, and 95% CI. The discriminative performance of brain MRI measures, plasma biomarkers, and MethylCog in classifying participants with MCI versus cognitively unimpaired status was evaluated using logistic regression models. For MRI measures, separate base models (demographics: age, sex, education) were extended with (a) total GM volume, (b) total WM volume, (c) total hippocampal volume, or (d) pWMH volume, followed by addition of MethylCog (base + MRI + MethylCog). For plasma biomarkers, the base model was extended with individual biomarkers or all biomarkers combined, and subsequently with MethylCog (base + biomarker[s] + MethylCog); sample sizes varied across models due to data availability. Regression coefficients, ORs, and 95% CIs were estimated for each model. Discriminative ability was quantified via the AUC, with nested model comparisons used to evaluate incremental changes in AUC upon inclusion of biomarkers/MRI or MethylCog.

## 3 RESULTS

### 3.1 Sample characteristics

As intended by the stratified sampling procedure, the training and test sets were comparable in age, education, sex distribution, Hispanic ethnicity, MCI prevalence, and APOE ε4 carrier status (all *p* > 0.05), although there was a modest difference in overall race distribution (*p* = 0.011), with the test set including a somewhat higher proportion of White participants and a lower proportion of participants in Other Race category. HBI participants were younger on average, more highly educated, and had a higher proportion of females and MCI cases than the HRS training and test samples. Relative to the full HRS sample aged 65 years and older, participants in the test set were slightly younger (75.20 vs. 75.96 years, *p* = 0.011) and had more years of education on average (12.89 vs. 12.63 years, *p* = 0.041), and differed modestly in race distribution (*p* < 0.001), with a higher proportion of White participants (84.8% vs. 77.7%) and lower proportions of Black/AA participants (12.4% vs. 16.5%) and the Other Race categories (2.8% vs. 5.8%). The samples did not differ significantly by sex (57.0% vs. 59.4%, *p* = 0.241) or Hispanic ethnicity (9.6% vs. 11.2%, *p* = 0.223). Relative to the full HBI cohort (*n* = 543), the subset with DNAm subset did not differ significantly in age (68.37 [10.44] vs. 68.5 [10.0] years, *p* = 0.904), education (16.21 [2.10] vs. 15.9 [2.4] years, *p* = 0.167), sex (69.6% vs. 68.6%, *p* = 0.814), or race (coded as White versus all other races) (85.7% vs. 78.1%, *p* = 0.069), but included fewer Hispanic participants (8.9% vs. 19.0%, *p* = 0.010) and a higher proportion of participants with MCI (33.0% vs. 26.5%, *p* = 0.025).

### 3.2 PCA of general cognitive ability

In the HRS‐HCAP, the first principal component (PC1) accounted for 41.6% of the variance across 19 cognitive measures (eigenvalue = 7.90). In HBI, PC1 accounted for 39.5% of the variance across 14 cognitive indicators (eigenvalue = 5.53). In HBI, g scores exhibited non‐normality and were standardized to mean 0 and SD 1 prior to subsequent analyses. Full methodological details are summarized in supplementary material including PCA loadings, scree plots (Figures S2 and S3), eigen values (Table S5 and S6), and imputation procedure for HBI cognitive tests (Table S7).

### 3.3 Model development, performance, and selection

**FIGURE 2:**
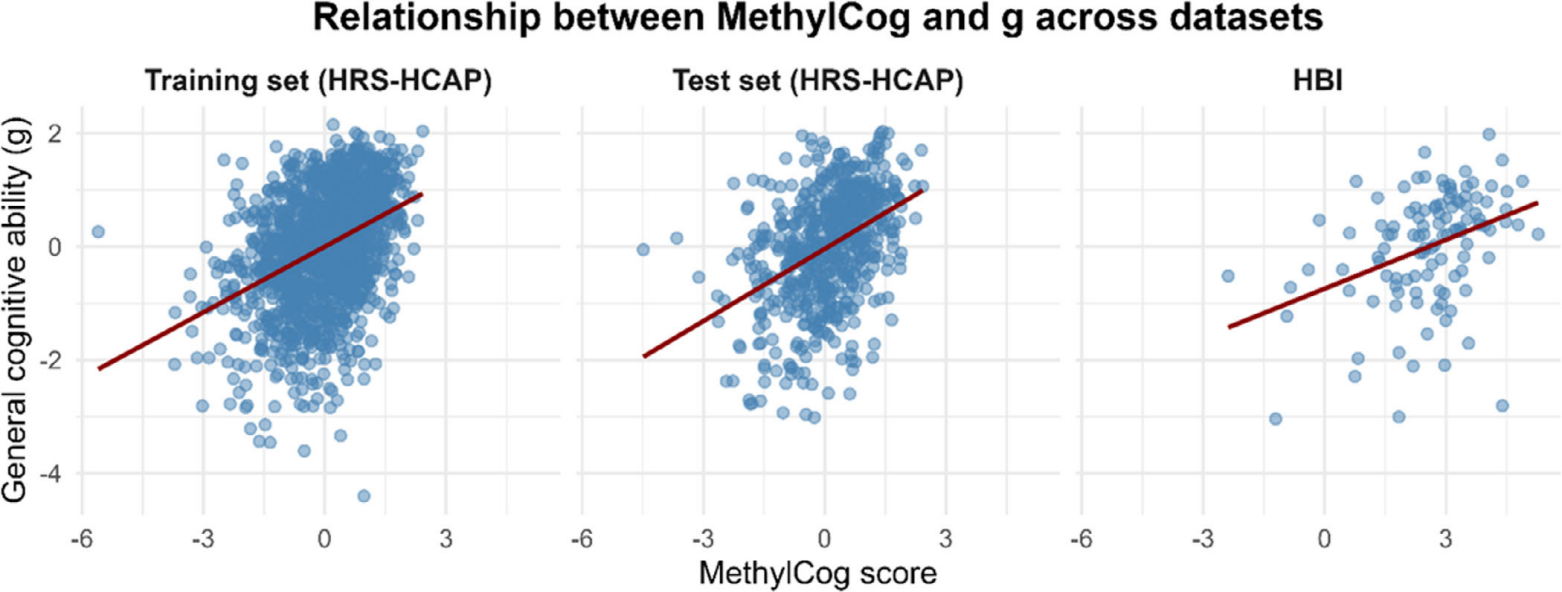
Correlations between MethylCog and measured general cognitive ability (g) in training, test, and HBI sets. HBI, Healthy Brain Initiative.

The joint cross‐validation procedure for the primary, batch‐adjusted elastic net model selected an α of 0.60 and a λ_1SE of 0.082, yielding a solution with 29 CpG probes assigned nonzero weights. In the HRS‐HCAP training set, this model achieved a cross‐validated mean squared error of 0.90 on standardized g. When applied to the HRS‐HCAP test set, MethylCog explained 17.0% of the variance in g (*R* ^2^ = 0.17; *r* = 0.41, *p* = 3.77 × 10^−26^; ρ = 0.43, *p* < 0.001). In the HBI set, MethylCog accounted for 14.2% of the variance in g (*R* ^2^ = 0.14; *r* = 0.38, *p* = 4.30 × 10^−5^; ρ = 0.40, *p* ≈ 1×10^−5^). Additional performance metrics are summarized in Table 2. Bivariate associations between MethylCog and g across all three sets (HBI set [*r* = 0.38, *p* < 0.001] and HRS‐HCAP test set[*r* = 0.41, *p* < 0.001]) are illustrated in Figure 2. Associations between individual CpG contributions showed broadly distributed effects, with a subset of loci (e.g., cg21609339, cg10313673) showing stronger associations with both MethylCog and g (Figure S4; Tables S8A, S8B, and Table S9).

**TABLE 2:** Performance metrics for the DNAm index of g.

Model	Alpha	Lambda	CpGs selected	Split	R^2^	RMSE	MAE	Pearson r	Spearman r
MethylCog	0.6	0.082	29	test	0.17	1.084	0.843	0.412	0.43
				HBI	0.14	1.112	0.835	0.376	0.38

*Note*: R^2^, RMSE, and MAE are computed on standardized scores (MethylCog) versus observed general cognitive ability. Pearson r and Spearman ρ are correlations with *p* values < 3.77 × 10^−26^ (TEST) and < 4.30 × 10^−5^ (HBI). Lambda is rounded to four decimal places. test = HRS test dataset; HBI = external validation dataset.

Abbreviations: HBI, Healthy Brain Initiative; HRS, Health and Retirement Study; MAE, mean absolute error; RMSE, root mean squared error.

### 3.4 Sensitivity analysis

Outlier exclusion did not meaningfully change the MethylCog—g correlation in either dataset (test set: *r* = 0.41 → 0.43; HBI: *r* = 0.38 → 0.30). MethylCog remained a significant predictor of g and MCI after adjusting for APOE ε4 carrier status (Table S10; all *p*s ≤ 0.033) and race/ethnicity (all *p*s ≤ 0.004). APOE ε4 moderated the MethylCog—cognition association in the HRS‐HCAP test set (β = 0.16, *p* = 0.046; Table S11), but corresponding interactions were not significant in HBI or for MCI. Race/ethnicity was not a significant moderator. Mediation analyses revealed that MethylCog partially mediated racial/ethnic group differences in cognition, accounting for 33%–45% of observed differences (both ACME *p*s ≤ 0.020; Table S12), although this effect was substantially attenuated after adjustment for education (HRS‐HCAP test set: 14.3%, *p* < 0.001; HBI: 39.4%, *p* = 0.066). To evaluate whether MethylCog's association with g was similar in participants without overt cognitive impairment only, analyses were repeated excluding MCI participants. In the HRS‐HCAP test set (*n* = 464), the association was comparable (*r* = 0.42, *p* < 0.001; Δ*R* ^2^ = 0.03). In HBI (*n* = 75), it was modestly attenuated but significant (*r* = 0.34, *p* = 0.003; Δ*R* ^2^ = 0.06, *p* = 0.021).

### 3.5 Differences in MethylCog by demographic and clinical characteristics

MethylCog scores were higher among cognitively unimpaired individuals and negatively associated with age in both cohorts. Scores differed by race group, with lower values in Black/African American and Other Race participants relative to White participants.; no significant differences were observed by sex or APOE ε4 status (Figures S5, S6, S7A, S7B, S8A, S8B, S9, S10, and S11; Table S13)

### 3.6 Criterion validity: variance explained in measured g

MethylCog showed incremental validity for measured g beyond demographic covariates (Table 3). In the test set, MethylCog was significantly associated with g beyond age and sex, increased explained variance from 19.1% to 30.1% (Δ*R* ^2^ = 0.11, *p* < 0.001). After additional adjustment for education, MethylCog remained a significant predictor, increasing explained variance from 43.6% to 46.9% (Δ*R* ^2^ = 0.03; estimate = 0.20, 95% CI 0.13–0.27, *p* < 0.001).

**TABLE 3:** Fully adjusted linear models predicting g from MethylCog and demographic covariates in HBI and the test sets.

Dataset	Variable	Estimate	SE	*t*	*p*	CI low	CI upper
HRS test	(Intercept)	1.915	0.357	5.372	<0.001	1.215	2.615
	Age	−0.049	0.004	−11.527	<0.001	−0.058	−0.041
	Sex	−0.186	0.061	−3.070	0.002	−0.305	−0.067
	Edu	0.145	0.011	13.685	<0.001	0.124	0.165
	MethylCog	0.201	0.033	6.061	<0.001	0.136	0.267
HBI	(Intercept)	−1.033	0.833	−1.239	0.218	−2.684	0.619
	Age	−0.020	0.008	−2.396	0.018	−0.037	−0.004
	Sex	−0.230	0.185	−1.244	0.216	−0.597	0.137
	Edu	0.118	0.042	2.826	0.006	0.035	0.201
	MethylCog	0.222	0.068	3.281	0.001	0.088	0.357

*Note*: Linear regression coefficients from models predicting general cognitive ability (g) in each cohort. All models include age, sex, years of education, and MethylCog. MethylCog is a DNA methylation—based cognition score (higher values indicate better cognitive functioning). *p* values are two‐tailed.

Abbreviations: CI, confidence interval; HBI, Healthy Brain Initiative; HRS, Health and Retirement Study; SE, standard error.

In HBI, MethylCog was significantly associated with g beyond age and sex, increasing explained variance from 6.7% to 17.5% (Δ*R* ^2^ = 0.11, *p* < 0.001). After further adjustment for education, MethylCog remained independently associated with g, increasing explained variance from 15.5% to 23.2% (Δ*R* ^2^ = 0.08; *B* = 0.22, 95% CI 0.09–0.36, *p* = 0.001).

### 3.7 Discriminatory ability of MethylCog for MCI

**FIGURE 3:**
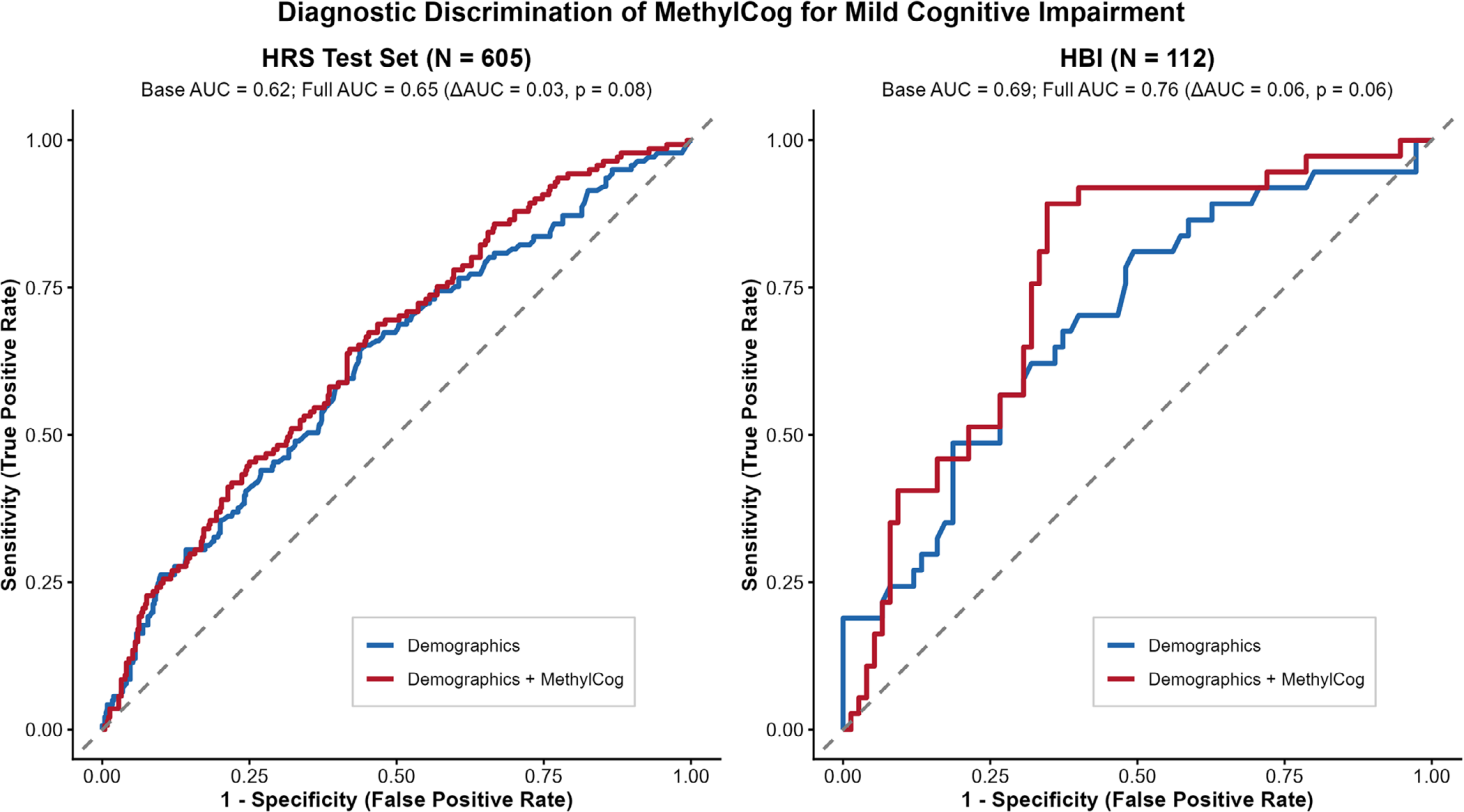
ROC curves comparing diagnostic discrimination of demographics‐only and demographics + MethylCog models for mild cognitive impairment. ROC curves show the diagnostic performance of logistic regression models for detecting mild cognitive impairment in two independent samples. The left panel shows results from the HRS Test Set (*N* = 605), while the right panel shows results from the HBI cohort (*N* = 112). Blue lines represent models using demographics alone (base model), and red lines represent models incorporating both demographics and MethylCog biomarkers (full model). The dashed diagonal line represents chance performance (AUC = 0.50). For the HRS Test Set, the base model achieved AUC = 0.62, and the full model achieved AUC = 0.65 (ΔAUC = 0.03, *p* = 0.08). For the HBI cohort, the base model achieved AUC = 0.69, and the full model achieved AUC = 0.76 (ΔAUC = 0.06, *p* = 0.06). AUC = area under the curve; HBI = Healthy Brain Initiative; HRS = Health and Retirement Study; ROC Receiver operating characteristic.

Higher MethylCog scores, were associated with reduced odds of MCI. In the HRS test set, each 1 SD increase in MethylCog was linked to a 26% reduction in MCI odds (OR = 0.74, 95% CI 0.60–0.92, *p* = 0.006) beyond age, sex, and education, with AUC increasing from 0.62 to 0.65 (ΔAUC = 0.03, *p* = 0.08; Figure 3). In HBI, a 1 SD higher MethylCog score was associated with a 33% reduction in MCI odds (OR = 0.67, 95% CI 0.46–0.94, *p* = 0.03), and AUC increased from 0.69 to 0.76 (ΔAUC = 0.06, *p* = 0.06).

### 3.8 Incremental value beyond cognitive screeners

MethylCog did not provide substantial incremental diagnostic value beyond brief cognitive screeners. In models including the MMSE (test set) or MoCA (HBI), which achieved good discrimination (AUCs = 0.77–0.89), adding MethylCog produced negligible AUC changes (ΔAUCs ≤ 0.001, both *p*s > 0.40) and was not independently associated with MCI diagnosis (ORs ≈ 0.85, *p*s > 0.19). Models are illustrated in Figure S12.

### 3.9 Associations with chronological age, GrimAge, and distinctness from epigenetic aging

MethylCog was negatively correlated with both chronological age (test set: *r* = −0.20, *p* < 0.001; HBI: *r* = −0.23, *p* = 0.014) and GrimAge (test: *r* = −0.35, *p* < 0.001; HBI: *r* = −0.32, *p* < 0.001; Figure S13 and Table S14) and GrimAge with chronological age was strongly positive as expected (tes: *r* = 0.77, *p* < 0.001; HBI: *r* = 0.91, *p* < 0.001). Partial correlations adjusting for chronological age showed that MethylCog's association with g remained significant in both cohorts (both *r* = 0.32, *p* < 0.001), as did the MethylCog—GrimAge (test set: *r* = −0.25, *p* < 0.001; HBI: *r* = −0.24, *p* = 0.011; Figure S14). Age‐residualized MethylCog (MethylCogResid) and GrimAge (GrimAgeResid) were modestly correlated (test set: *r* = −0.30; HBI: *r* = −0.29; both *p*s ≤ 0.002), indicating partial overlap but not redundancy. MethylCogResid showed stronger and more consistent associations with g than GrimAgeResid (test: *r* = 0.33 vs. *r* = −22; HBI: *r* = 0.32, *p* = 0.001 vs. *r* = −11, *p* = 0.23).

Hierarchical linear models (Table 4) showed that MethylCogResid explained more incremental variance in g than GrimAgeResid. In the test set, adding GrimAgeResid to demographics increased *R* ^2^ by ∼0.02 (sr^2^ = 0.019), with MethylCogResid contributing a further ∼0.02 (sr^2^ = 0.022). In HBI, GrimAgeResid added negligibly (Δ*R* ^2^ ≈ 0.01, *p* = 0.88), whereas MethylCogResid substantially improved fit (Δ*R* ^2^ ≈ 0.07, *p* = 0.003).

**TABLE 4:** Hierarchical linear model predicting g from GrimAgeResid and MethylCogResid, adjusted for demographic covariates.

Dataset	Predictor	B	SE	t	*p*	sr^2^
HRS test	Intercept	2.343	0.358	6.54	<0.001	
	Age	−0.055	0.004	−13.08	<0.001	
	Sex	−0.116	0.065	−1.78	0.075	
	Education	0.143	0.011	13.58	<0.001	
	GrimAgeResid	−0.100	0.034	−2.98	0.003	0.019
	MethylCogResid	0.164	0.033	4.98	0.000	0.022
HBI	Intercept	−0.033	0.822	−0.04	0.968	
	Age	−0.027	0.008	−3.25	0.002	
	Sex	−0.248	0.207	−1.20	0.234	
	Education	0.120	0.042	2.84	0.005	
	GrimAgeResid	0.015	0.099	0.16	0.876	0.006
	MethylCogResid	0.283	0.092	3.09	0.003	0.069

*Note*: Values are unstandardized coefficients from linear regression models predicting general cognitive ability (g). Separate models were fit in the HRS Test and HBI cohorts, each adjusted for age, sex, and education. GrimAgeResid and MethylCogResid are residualized scores obtained by regressing GrimAge and MethylCog, respectively, on chronological age, such that higher GrimAgeResid indicates more accelerated epigenetic aging and higher MethylCogResid indicates better‐than‐expected DNAm‐derived cognitive capacity for age. B = unstandardized regression coefficient; sr^2^ = semi‐partial $R^{2}$ representing the unique proportion of variance in g explained by the predictor, controlling for all other variables in the model. Statistical significance is based on two‐tailed tests.

Abbreviations: HBI, Healthy Brain Initiative; HRS, Health and Retirement Study; SE, standard error.

Logistic regression models (Table 5) showed a similar pattern. GrimAgeResid was not significantly associated with MCI in either cohort (test: OR = 1.19, *p* = 0.10; HBI: OR = 1.48, *p* = 0.14). MethylCogResid was associated with lower odds of MCI in the test set (OR = 0.79, *p* = 0.031) and showed a similar but trend‐level association in HBI (OR = 0.66, *p* = 0.077). AUC improvements from adding both residualized measures were modest and did not reach significance in either cohort (test set: base AUC = 0.62 → 0.65; HBI: 0.69 → 0.77). Overall, MethylCogResid showed a more consistent association with cognition and impairment than GrimAgeResid, although incremental classification accuracy remained modest.

**TABLE 5:** Logistic regression predicting mild cognitive impairment from GrimAgeResid and MethylCogResid in the test and HBI sets.

Dataset	Predictor	B_logit	SE	z	*p*	OR	OR_CI_lo	OR_CI_hi
Test set	Intercept	−4.915	1.17	−4.202	<0.001	0.007	0.001	0.071
	Age	0.055	0.014	3.975	<0.001	1.056	1.028	1.085
	Sex	−0.125	0.218	−0.573	0.566	0.883	0.575	1.35
	Education	−0.031	0.034	−0.936	0.349	0.969	0.908	1.036
	GrimAgeResid	0.177	0.108	1.639	0.101	1.194	0.964	1.475
	MethylCogResid	−0.231	0.107	−2.158	0.031	0.794	0.644	0.98
HBI	Intercept	−2.108	2.073	−1.016	0.309	0.122	0.002	6.855
	Age	0.066	0.023	2.828	0.005	1.069	1.022	1.122
	Sex	0.241	0.534	0.451	0.652	1.272	0.437	3.601
	Education	−0.207	0.107	−1.927	0.054	0.813	0.654	1
	GrimAgeResid	0.393	0.263	1.493	0.135	1.481	0.891	2.522
	MethylCogResid	−0.417	0.236	−1.77	0.077	0.659	0.404	1.034

*Note*: Values represent coefficients from logistic regression models predicting mild cognitive impairment (1) versus cognitively unimpaired status (0). All models adjusted for age, sex, and education. GrimAgeResid and MethylCogResid reflect residualized versions of GrimAge and MethylCog after removing chronological age to isolate age‐independent variance. $B_{\mathrm{logit}}$ = log‐odds coefficient; 95% confidence intervals for the OR are shown in the OR_CI columns. ORs greater than 1 indicate increased odds of impairment; ORs less than 1 indicate reduced odds. For the test set, higher MethylCogResid was significantly associated with lower odds of impairment, whereas GrimAgeResid was not significantly related to impairment. In HBI, both predictors showed similar directional patterns, although confidence intervals overlapped 1. Statistical significance is based on two‐tailed tests. *p* < 0.05 is denoted in text.

Abbreviations: CI, confidence interval; HBI, Healthy Brain Initiative; OR, odds ratio.

### 3.10 Exploratory associations with ADRD biomarkers and MRI (HBI only)

MethylCog showed no significant age‐ and sex‐adjusted partial correlations with ADRD plasma biomarkers or MRI measures (all |r| ≤ 0.11, all *p*s ≥ 0.28). By contrast, measured g showed small but significant associations with log‐transformed pTau181 (r_partial = −0.20, *p* = 0.040) and periventricular WMH burden (r_partial = −0.22, *p* = 0.038); partial correlations of g with remaining biomarkers and MRI volumes were non‐significant (all |r| ≤ 0.18, all *p*s ≥ 0.08; Figure S15). Given the modest sample size and absence of dementia cases, these null associations should be interpreted as preliminary.

In logistic regression models predicting MCI, individual plasma biomarkers added minimally to demographic base models (ΔAUCs ≤ 0.01, all *p*s ≥ 0.13), and none were independently associated with diagnosis (ORs near 1.0, all *p*s > 0.30). By contrast, MethylCog was significantly associated with lower odds of MCI across all biomarker‐adjusted models (ORs = 0.64–0.68, *p*s ≈ 0.02–0.04), achieving AUCs of 0.75–0.77 that were essentially indistinguishable from MethylCog‐only models, suggesting MethylCog performed at least as well as the individual biomarkers examined (Figure S16). For MRI, lower GM and hippocampal volumes were associated with higher MCI odds (GM OR ≈ 0.97, *p* ≈ 0.03), with MRI‐only models yielding AUCs of 0.72–0.78. Adding MethylCog to MRI models increased AUCs to approximately 0.79–0.80, with the largest gain observed for the periventricular WMH model (AUC: 0.74 → 0.80; Figure S17). These exploratory comparisons should be interpreted cautiously given the limited sample size.

## 4 DISCUSSION

In the held‐out test set, MethylCog accounted for 17% of the variance in measured g; in an external validation set (HBI), it accounted for 13%. Beyond age and sex, MethylCog explained an additional ∼11% of the variance in g in both cohorts. MethylCog showed consistent associations with cognitive diagnosis over and above demographic factors but did not confer additional discriminative value once brief cognitive screeners (MMSE/MoCA) were included. Comparisons with DNAm GrimAge suggest that MethylCog captures a cognition‐specific methylation signal only partly related to epigenetic aging, and exploratory analyses in HBI indicated no significant associations with AD plasma biomarkers or structural MRI measures.

These findings extend prior work on DNAm and cognition. McCartney and colleagues showed that a high‐dimensional DNAm predictor explained ∼4% of the variance in g beyond age and sex in external cohorts.^26^ MethylCog's incremental contribution beyond age and sex was approximately two to three times larger, despite using only 29 probes, though differences in cohort composition, cognitive batteries, and analytical approaches preclude direct comparison. Nonetheless, that a parsimonious, EWAS‐informed model achieves comparable or greater prediction with a fraction of the probes supports the feasibility of targeted DNAm scores for cognitive phenotyping. These findings also replicated in an independent cohort of a more modest sample size (i.e., 112 participants), supporting the portability of the score.

Functional annotation of the 29 CpGs offers preliminary biological context. The retained loci map to genes implicated in synaptic signaling and plasticity (e.g., PPP1CA, CAMTA2, SLC12A5), innate immune and inflammatory regulation (e.g., SOCS3, BCL3, AIM2), and cellular maintenance and metabolic stability (e.g., CD81, INPP5A, POLK). The direction of coefficients broadly aligns with expected biological roles; promoter hypomethylation in several synaptic genes may reflect upregulation of pathways supporting neurotransmission, whereas methylation in immune‐related gene bodies may index regulatory processes that mitigate age‐related inflammatory burden. That said, elastic net selection optimizes prediction rather than isolating causal loci, peripheral blood methylation may not recapitulate brain‐specific epigenetic patterns, and interpreting individual CpG effects within a composite score requires caution. These annotations are best understood as a foundation for targeted mechanistic inquiry rather than as evidence of specific regulatory mechanisms.

The magnitude and pattern of effects are also informative when considered alongside established epigenetic clocks. Consistent with prior work, GrimAge showed strong associations with chronological age,^14^, ^15^ whereas the age‐unadjusted MethylCog had only modest, negative correlations with age, suggesting that the latter reflects more than a generic aging signal. After residualizing both measures for chronological age, MethylCogResid showed stronger and more consistent associations with general cognition and MCI than GrimAgeResid in both cohorts. In the heldout HRS‐HCAP sample, MethylCogResid explained more unique variance in cognition than GrimAgeResid, and in HBI GrimAgeResid was not significantly related to cognition whereas MethylCogResid remained robustly associated. Together, these patterns suggest that MethylCog is not simply a repackaged aging clock, but instead captures methylation variation more tightly linked to cognitive performance per se. This aligns with theoretical models positing partially separable methylation signatures for systemic biological aging versus cognition‐relevant neural and vascular processes.^26^, ^61^

### 4.1 MethylCog as a proxy of individual differences in cognitive ability

Several lines of evidence converge on the interpretation that MethylCog primarily indexes stable, trait‐like variation in cognitive ability, possibly reflecting the cumulative influence of environmental factors on the methylome, rather than variance attributable to active neurodegenerative disease. MethylCog was developed in a sample from which individuals with dementia were excluded and validated in a cohort without dementia cases, a design intended to isolate cognitive variation in the absence of overt pathology. Sensitivity analyses restricting to cognitively unimpaired participants further showed that MethylCog's association with g was not contingent on the inclusion of individuals with MCI, consistent with a measure that tracks continuous variation within the normative range.

The pattern of covariate associations supports this interpretation. Education is among the strongest determinants of cognitive performance and serves as a proxy for cumulative environmental enrichment, occupational complexity, and access to health‐promoting resources.^62^ When education was included as a covariate, MethylCog's association was attenuated yet remained significant, consistent with shared variance between educational attainment and cognition‐related DNAm.^18^, ^63^, ^64^ This overlap is expected, as DNAm is influenced by many of the same developmental, socioeconomic, and behavioral exposures that education indexes;^63^, ^65^, ^66^, ^67^ However, MethylCog's retention of independent signal suggests it also indexes biological variation not fully accounted for by this proxy. The mediation analyses extend this logic. Racial/ethnic minority participants had lower MethylCog scores in both cohorts, and MethylCog partially mediated racial/ethnic differences in cognitive performance (approximately 33%–45% of total effects, 14%–39% after education adjustment). These group differences are more parsimoniously understood as reflecting cumulative social and environmental exposures rather than inherent biological differences. Socioeconomic disadvantage has been associated with differential DNAm in stress‐ and inflammation‐related genes^19^ and with accelerated epigenetic aging^18^, ^68^ and racial discrimination has been linked to epigenetic age acceleration.^69^ The observed mediation pattern is consistent with these frameworks but should be considered preliminary given limited racial/ethnic diversity and sample sizes.

Associations with ADRD biomarkers, MRI measures, and cognitive screeners are also interpretable within this framework, though all remain exploratory given HBI's limited sample size and absence of dementia cases. MethylCog showed no significant associations with ADRD blood biomarkers or MRI measures, whereas measured g showed small associations with pTau181 and periventricular WMH. Measured cognitive performance integrates both premorbid capacity and concurrent disease‐related decrements, while ADRD biomarkers primarily reflect active pathology.^70^, ^71^ The absence of MethylCog—biomarker associations in this low‐pathology sample is consistent with a measure that captures the trait‐like component of cognitive ability rather than disease‐related variance, though this interpretation requires confirmation in cohorts with greater pathological burden.

Similarly, MethylCog added no discriminative value beyond cognitive screeners, which capture both trait‐level ability and pathology‐related decline,^72^ yet improved MCI discrimination beyond demographics alone, consistent with the premise that an individual's level of cognitive ability influences who crosses the threshold to clinical impairment.^71^, ^72^, ^73^ Whether MethylCog remains independent of disease markers across the AD continuum, and whether DNAm‐based cognitive indices are indeed stable or also sensitive to preclinical neurodegenerative change, are questions that will require larger, more clinically diverse samples and longitudinal repeated‐measures designs.

The present work has several implications for future research. First, the approach of leveraging existing EWAS findings with penalized regression in a well‐phenotyped cohort could be extended to domain‐specific cognitive factors or composite multi‐omic scores. Second, the moderate variance explained provides a starting point for power calculations in larger consortia. Third, MethylCog's parsimonious probe set makes it amenable to targeted assays, facilitating testing in large‐scale epidemiological research. Whether DNAm‐based indices of cognitive ability also prove informative alongside disease biomarkers in understanding heterogeneity in cognitive trajectories remains an open question. Individuals with comparable AD biomarker burden may differ in their rate of cognitive decline as a function of their underlying cognitive ability; a molecular measure of that ability could complement disease markers without itself serving as a disease marker. Prospective designs with incident diagnostic outcomes would help evaluate this possibility.

## 5 LIMITATIONS

Several limitations should be acknowledged. The external validation cohort was modest in size with more years of education on average than the HRS‐HCAP sets, which may limit generalizability. The candidate CpG approach may have missed informative loci that would emerge in a genome‐wide model. We focused on a single epigenetic clock comparator, GrimAge; other DNAm aging measures may offer complementary information. Additionally, the training sample included individuals with MCI, some of whom may harbor underlying pathology, and MethylCog could partly reflect disease‐related epigenetic alterations. Finally, disentangling trait‐level cognitive variation from preclinical change will require longitudinal designs with repeated DNAm and biomarker assessment.

## 6 CONCLUSION

In summary, this study provides initial evidence that a parsimonious DNAm proxy for general cognitive ability, derived from prior EWAS CpGs, serves as a modest but replicable marker of individual differences in cognitive ability in older adults. MethylCog replicated across independent cohorts, captured information only partly overlapping with epigenetic aging clocks, and showed no detectable associations with AD biomarkers in exploratory analyses, a pattern consistent with a score that reflects trait‐like cognitive ability rather than active disease processes. In research contexts where direct cognitive assessment is unavailable, such as retrospective biospecimen analyses, biobank studies, or life‐course epidemiology, MethylCog could provide a scalable, biologically grounded proxy for cognitive functioning. Future work in larger, longitudinal, and more diverse cohorts is needed to determine whether MethylCog and related scores can inform cognitive trajectories, complement disease biomarkers, and contribute to multimodal approaches for understanding cognitive aging and dementia risk.

## CONFLICT OF INTEREST STATEMENT

The authors declare that they have no conflicts of interest related to the content of this manuscript. Author disclosures are available in the supporting information.

## CONSENT STATEMENT

All participants provided written informed consent prior to participation in the study under a Certificate of Confidentiality from the National Institutes of Health.

## Supporting information




**Supporting Information**: alz71421‐sup‐0001‐tableS3.xlsx

**Supporting Information**: alz71421‐sup‐0002‐SuppMat.docx

**Supporting Information**: alz71421‐sup‐0003‐SuppMat.pdf
