# Supplementary material for: Development and validation of MethylCog, a blood DNA methylation proxy for cognition
Source: Alzheimers Dement. 2026 Apr 30;22(5):e71421. doi: 10.1002/alz.71421 (PMC13132682; doi:10.1002/alz.71421)
Supplement: Supplementary file 2 — Supporting Information: alz71421‐sup‐0002‐SuppMat.docx [file ALZ-22-e71421-s001.docx]

**Supplementary Material**

**HRS-HCAP Cognitive Measures**

**Supplementary** **Table 1.** provides detailed descriptions of all cognitive tests included in the HRS-HCAP PCA and associated factor loadings. For each measure, the table reports the cognitive domain, task description, and its loading on the general cognition factor (g).

**Table S1.** Cognitive Measures and Factor Loadings for the HRS–HCAP General Cognitive Factor

| **Construct / Domain** | **Measure** | **Description** | **Factor Loading** |
| --- | --- | --- | --- |
| **Episodic Memory** | Brave Man Story – Immediate Recall | Immediate recall of a 3-sentence story from the East Boston Memory Test[1]; score reflects number of correct details recalled on each trial. | **0.520** |
|  | Logical Memory I – Immediate Recall | Logical Memory I from the Wechsler Memory Scale;[2] score reflects number of story units correctly recalled. | **0.678** |
|  | CERAD Word List – Immediate Total | Immediate recall across three learning trials of a 10-item word list from the Consortium to Establish a Registry for Alzheimer’s Disease (CERAD) battery [3]; | **0.698** |
|  | Brave Man Story – Delayed Recall | Delayed recall of the story from the East Boston Memory Test; score reflects number of details recalled after delay. | **0.557** |
|  | Logical Memory II – Delayed Recall | Delayed narrative recall from the Wechsler Memory Scale; score reflects number of story units recalled after delay. | **0.705** |
|  | CERAD Word List – Delayed Recall | Delayed free recall of the 10-word CERAD list. | **0.696** |
|  | CERAD Constructional Praxis – Delayed Recall | Delayed reproduction of four geometric figures assessing visuoconstructional memory. | **0.623** |
|  | CERAD Word List – Recognition | Word recognition accuracy (correct recognitions minus false positives). | **0.518** |
| **Attention / Working Memory / Processing Speed** | Backward Counting | Timed backwards counting from the Brief Test of Adult  Cognition by Telephone; count backward from 100 for 30 seconds (BTACT).[4] | **0.630** |
|  | Symbol Digit Modalities Test[5] | Processing speed, attention, and working memory; total number of correct digit–-symbol pairings in 90 seconds. | **0.814** |
|  | Letter Cancellation Task | A letter cancellation task developed by the National (UK) Survey of Health and Development.[6]Visual search and sustained attention; speed and accuracy scored from scanning target letters. | **0.596** |
| **Executive Function** | Trail Making Test – Part A[7] | Processing speed and visual attention; completion time reverse-coded so higher scores indicate better performance. | **0.650** |
|  | Trail Making Test – Part B[7] | Set-shifting and divided attention; completion time reverse-coded. | **0.649** |
| **Fluid Reasoning** | Raven’s Standard Progressive Matrices (adapted version)[8] | Nonverbal reasoning and fluid intelligence; total correct responses to geometric pattern completion items. | **0.709** |
|  | HRS Number Series[9] | Inductive reasoning; score reflects accuracy in identifying numeric patterns. | **0.695** |
| **Language / Semantic Memory** | Animal Naming (Semantic Fluency) adapted from Woodcock Johnson-III Tests of Achievement[10] | Total number of animals named in 60 seconds; assesses semantic memory and processing speed. | **0.640** |
| **Visuoconstruction** | CERAD Constructional Praxis - Copy[3] | Immediate copy of four geometric figures assessing visuoconstructional ability; maximum score 11. | **0.516** |
| **Global Cognition** | Mini-Mental State Examination Total Score[11] | Global cognitive screening measure assessing orientation, attention, memory, language, and visuoconstruction; total score 0–30. | **0.700** |

**HBI Cognitive Measures**

**Supplementary Table 2.** HBI Cognitive Measures and Corresponding Factor Loadings

| **Construct / Domain** | **Measure** | **Description** | **Factor Loading** |
| --- | --- | --- | --- |
| **Global Cognition** | Montreal Cognitive Assessment (MoCA Total)[12] | Global cognitive screening measure assessing multiple domains including memory, executive function, visuospatial abilities, language, attention, and orientation; scores range 0–30. | **0.350** |
| **Episodic Memory** | Hopkins Verbal Learning Test – Revised; Immediate Recall (HVLT Immediate)[13] | Immediate recall across three learning trials of a 12-word list; total possible = 36. | **0.299** |
|  | Craft Story – Immediate Recall (CS Immediate Recall)[14] | Immediate recall of a short narrative story; scored as story units (0–25) or verbatim details (0–44). | **0.260** |
|  | Hopkins Verbal Learning Test – Delayed Recall (HVLT Delayed) | Delayed free recall of the 12-word list following a time delay. | **0.322** |
|  | Craft Story – Delayed Recall (CS Delayed Recall) | Delayed narrative recall after ~20 minutes; scored as story units or verbatim details. | **0.230** |
|  | Benson Figure Recall – Delayed Recall[15] | Delayed reproduction of a complex geometric figure; assesses visual memory; max score = 17. | **0.246** |
|  | Hopkins Verbal Learning Test – Recognition Discrimination (HVLT Recognition) | Recognition memory accuracy (correct recognitions minus false positives). | **0.178** |
| **Attention / Working Memory** | Digit Span Forwards Wechsler Adult Intelligence Scale ( WAIS IV)[16] – Forward | Auditory attention; longest digit sequence repeated correctly (0–9). | **0.214** |
|  | Digit Span – Backward | Auditory working memory; longest sequence repeated in reverse (0–8). | **0.176** |
| **Processing Speed / Working Memory** | Number Symbol Coding[17] | Processing speed, attention, and working memory; number of correct symbol–digit substitutions in 90 seconds (max = 70). | **0.313** |
| **Executive Function** | Trail Making Test – Part A (reverse-coded time)[7] | Processing speed and visual attention; completion time reverse-coded so higher values = better performance. | **0.265** |
|  | Trail Making Test – Part B (reverse-coded time)[7] | Set-shifting and divided attention; time reverse-coded. | **0.316** |
| **Language / Semantic Memory** | Animal Naming (Semantic Fluency)[10] | Number of animals named in 60 seconds; assesses semantic retrieval and processing speed. | **0.261** |
| **Confrontation Naming** | Multilingual Naming Test (MINT Total)[18] | Confrontation naming of 32 pictured objects; total correct responses scored. | **0.243** |

**Final Elastic net model to derive MethylCog**. Elastic-net regression coefficients (β) for the 29 CpG sites retained in the final MethylCog predictor, beta weights shown in **Supplementary Table 4**. **Supplementary Figure 1** show bars represent standardized coefficients from the optimal λ selected via 10-fold cross-validation. Positive values (teal) indicate higher methylation associated with better cognitive function; negative values (red) indicate the opposite. CpGs are ordered by the absolute magnitude of their coefficients. Gene annotations appear in parentheses.


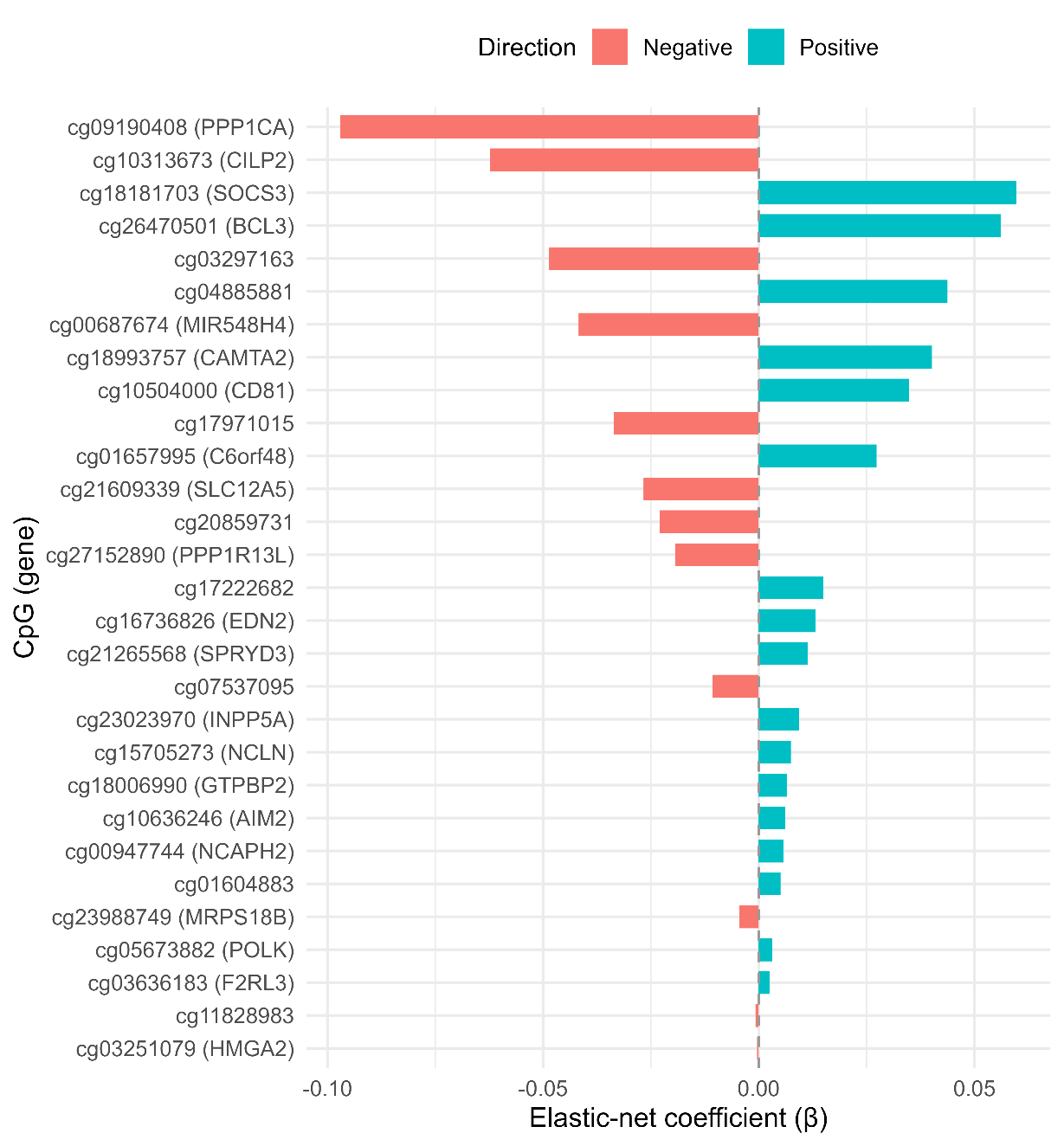


**Supplementary Figure 1. Final CpG weights selected from elastic net**

**Supplementary Table 4. Final CpG weights selected from elastic net that compose MethlyCog**

| CpG | Beta |
| --- | --- |
| cg09190408 | -0.09701 |
| cg10313673 | -0.06224 |
| cg18181703 | 0.059794 |
| cg26470501 | 0.056132 |
| cg03297163 | -0.04859 |
| cg04885881 | 0.043786 |
| cg00687674 | -0.04173 |
| cg18993757 | 0.040159 |
| cg10504000 | 0.034848 |
| cg17971015 | -0.03361 |
| cg01657995 | 0.027307 |
| cg21609339 | -0.0267 |
| cg20859731 | -0.02295 |
| cg27152890 | -0.01945 |
| cg17222682 | 0.014953 |
| cg16736826 | 0.013178 |
| cg21265568 | 0.011415 |
| cg07537095 | -0.01072 |
| cg23023970 | 0.009351 |
| cg15705273 | 0.00745 |
| cg18006990 | 0.006597 |
| cg10636246 | 0.006118 |
| cg00947744 | 0.005685 |
| cg01604883 | 0.005064 |
| cg23988749 | -0.00447 |
| cg05673882 | 0.003167 |
| cg03636183 | 0.00247 |
| cg11828983 | -0.00073 |
| cg03251079 | -0.00041 |

**Principal Component Analysis (PCA)**

**HRS–HCAP** PCA was conducted on 19 cognitive indicators from 2,069 participants with complete data for DNA methylation analyses. Non-completion codes for Trail Making Test A and B were recoded as missing and imputed using variable-wise predictive mean matching (m = 1; 5 iterations). PC1 had an eigenvalue of 7.90, explaining **41.6%** of variance (**Supplementary Table 5)**. PC2 explained 10.2%, with all additional components <6%. A clear elbow was observed after PC1 (**Figure 2**). Loadings were uniformly positive and moderate-to-high (**Supplementary Table 1**), consistent with a strong general cognitive factor. Highest loadings included: Digit Span (0.81)**,** Logical Memory delayed recall (0.71)**,** Category fluency (0.71)**,** MMSE total (0.70)**,** CERAD Word List immediate recall (0.70)**,** Word list recognition (0.70)**,** Number Series (0.70)**.** Reverse-coded Trail Making Tests A and B also loaded strongly (~0.65). PC1 scores were extracted using regression-based scoring, with higher scores reflecting better global cognition.

**HBI PCA** PCA was conducted on 14 cognitive tests from 112 HBI participants. Missing values (67/1,568 = 4.27%) were imputed using variable-wise median imputation (details in **Supplementary Table 7**). PC1 had an eigenvalue of 5.53, accounting for **39.5%** of variance (**Supplementary Table 6)**. PC2 explained 14.8%; all subsequent components explained <9% (**Figure 3**).Top PC1 loadings included: MoCA total (0.35), HVLT delayed recall (0.33), TMT-B (reverse-scored; 0.32), Number–Symbol Coding (0.31), HVLT immediate recall (0.30), Animal fluency (0.27), TMT-A (reverse-scored; 0.26). PC1 scores were inverted (so higher = better cognition) and standardized (mean 0, SD 1). The g-factor correlated strongly with MoCA (r = 0.82), supporting construct validity.

**Supplementary Table 5.** Eigenvalues and Variance Explained for the HRS–HCAP Principal Components Analysis

| **Component** | **Eigenvalue** | **Proportion of Variance** | **Cumulative Variance** |  |
| --- | --- | --- | --- | --- |
| PC1 | 7.900 | 0.416 | 0.416 |  |
| PC2 | 1.929 | 0.102 | 0.517 |  |
| PC3 | 1.089 | 0.057 | 0.575 |  |
| PC4 | 1.042 | 0.055 | 0.629 |  |
| PC5 | 0.871 | 0.046 | 0.675 |  |
| PC6 | 0.731 | 0.038 | 0.714 |  |
| PC7 | 0.700 | 0.037 | 0.751 |  |
| PC8 | 0.554 | 0.029 | 0.780 |  |
| PC9 | 0.541 | 0.028 | 0.808 |  |
| PC10 | 0.529 | 0.028 | 0.836 |  |
| PC11 | 0.479 | 0.025 | 0.861 |  |
| PC12 | 0.462 | 0.024 | 0.886 |  |
| PC13 | 0.422 | 0.022 | 0.908 |  |
| PC14 | 0.400 | 0.021 | 0.929 |  |
| PC15 | 0.389 | 0.020 | 0.949 |  |
| PC16 | 0.352 | 0.019 | 0.968 |  |
| PC17 | 0.240 | 0.013 | 0.981 |  |
| PC18 | 0.206 | 0.011 | 0.991 |  |
| PC19 | 0.162 | 0.009 | 1.000 |  |

Note. Eigenvalues, proportion of variance explained, and cumulative variance are based on an unrotated principal components analysis of 19 cognitive indicators from the HRS–HCAP cohort (N = 2,069). PC1 was extracted as the general cognitive factor (g-factor).

**Supplementary Table 6.**  Eigenvalues and Variance Explained for the HBI Principal Components Analysis

| **Component** | **Eigenvalue** | **Proportion of Variance** | **Cumulative Variance** |
| --- | --- | --- | --- |
| PC1 | 5.429 | 0.388 | 0.388 |
| PC2 | 1.999 | 0.143 | 0.531 |
| PC3 | 1.169 | 0.083 | 0.614 |
| PC4 | 1.102 | 0.079 | 0.693 |
| PC5 | 0.753 | 0.054 | 0.747 |
| PC6 | 0.736 | 0.053 | 0.799 |
| PC7 | 0.597 | 0.043 | 0.842 |
| PC8 | 0.472 | 0.034 | 0.876 |
| PC9 | 0.426 | 0.030 | 0.906 |
| PC10 | 0.389 | 0.028 | 0.934 |
| PC11 | 0.352 | 0.025 | 0.959 |
| PC12 | 0.243 | 0.017 | 0.976 |
| PC13 | 0.209 | 0.015 | 0.991 |
| PC14 | 0.124 | 0.009 | 1.000 |

**Note. Eigenvalues, proportion of variance explained, and cumulative variance are based on an unrotated principal components analysis of 14 cognitive indicators from the HBI external validation dataset (N = 112). PC1 was extracted as the general cognitive factor (g-factor). Values are rounded to three decimals for clarity.**


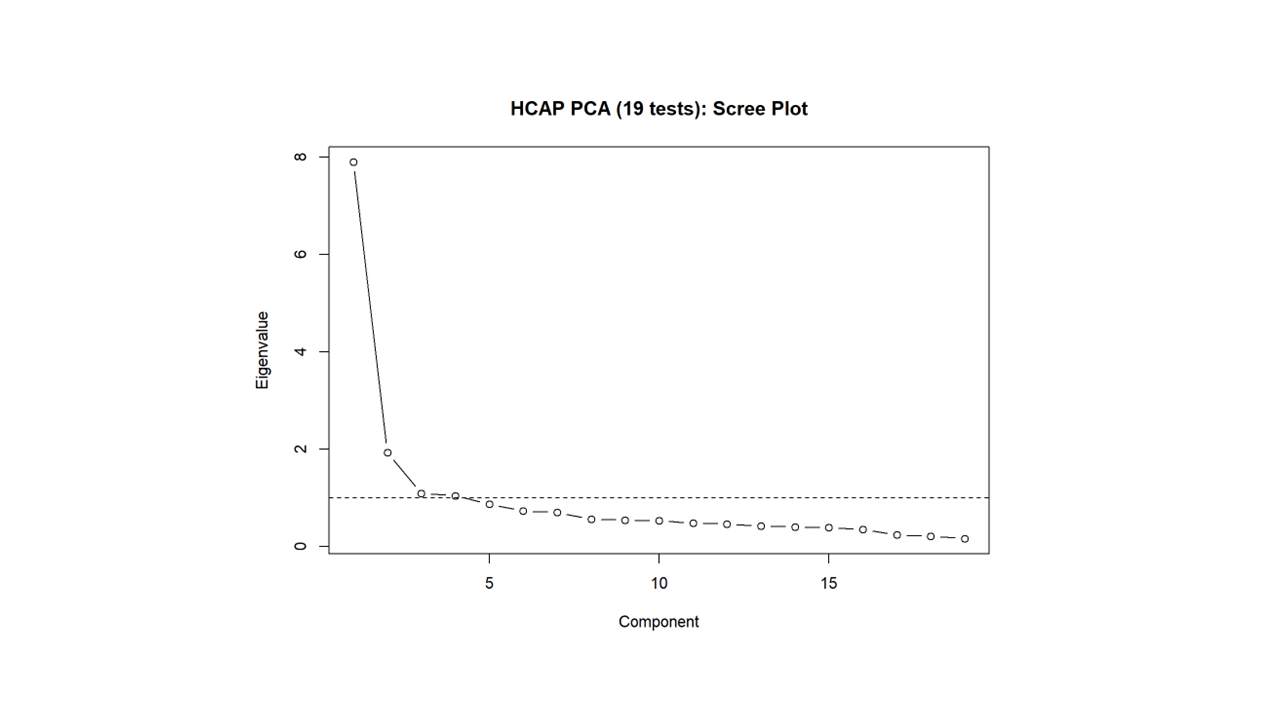


**Supplementary Figure 2.**  Scree plot for the HRS–HCAP principal components analysis (19 cognitive tests)

Note. The plot displays eigenvalues for each principal component extracted from the HRS–HCAP cognitive test battery. The sharp decline from PC1 to PC2, followed by a clear elbow after the first component, indicates that PC1 accounts for a disproportionately large amount of variance relative to subsequent components. The dashed horizontal line denotes the Kaiser criterion (eigenvalue = 1).


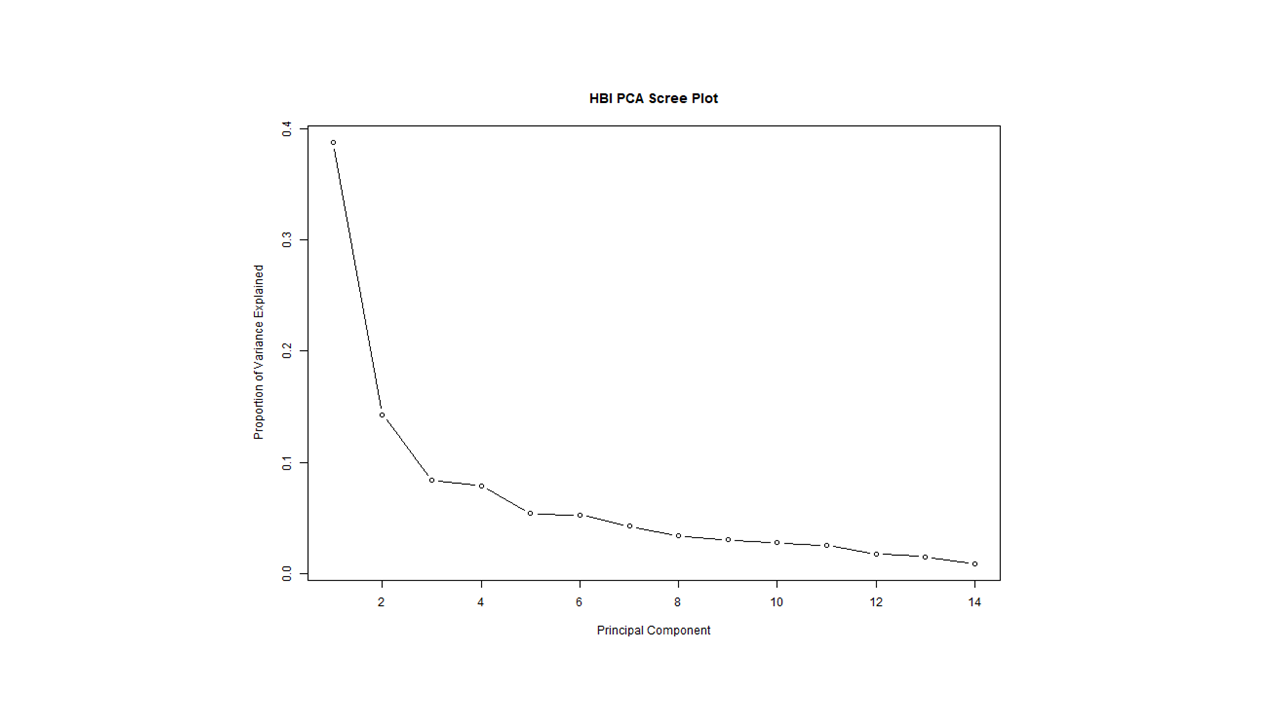


**Supplementary Figure 3.** Scree plot for the HBI principal components analysis (14 cognitive tests). Note. The scree plot shows the proportion of variance explained by each principal component in the HBI cognitive test battery. PC1 explains the largest share of variance (≈39%), followed by a steep decline to PC2 and a clear leveling-off thereafter, indicating an “elbow” pattern consistent with retaining only the first component. This supports interpretation of PC1 as a general cognitive ability factor (g-factor), analogous to the solution obtained in HRS–HCAP.

**Supplementary Table 7**. Missing Data and Imputation Summary for Cognitive Measures in the HBI Dataset

| **Variable** | **N Imputed** | **Percent Imputed** |
| --- | --- | --- |
| Montreal Cognitive Assessment Total | 3 | 2.68% |
| HVLT–Revised Immediate Recall | 4 | 3.57% |
| HVLT–Revised Delayed Recall | 4 | 3.57% |
| HVLT–Revised Recognition Discrimination | 4 | 3.57% |
| Benson Figure Delayed Recall | 6 | 5.36% |
| Craft Story Immediate Recall | 6 | 5.36% |
| Craft Story Delayed Recall | 6 | 5.36% |
| Trail Making Test Part A, reverse-coded | 4 | 3.57% |
| Trail Making Test Part B, reverse-coded | 5 | 4.46% |
| Number–Symbol Coding | 4 | 3.57% |
| Animal Naming – Semantic Fluency | 4 | 3.57% |
| Multilingual Naming Test | 9 | 8.04% |
| Digit Span Forward | 4 | 3.57% |
| Digit Span Backward | 4 | 3.57% |

Note. Missing values were imputed using variable-wise median imputation before conducting principal components analysis (PCA). Across all HBI cognitive measures (N = 112 participants), 67 of 1,568 total values (4.27%) were imputed. Percent imputed reflects the proportion of missing observations for each variable.

**Bivariate CpG Associations with MethylCog and g** Pearson correlations between each CpG, the MethylCog score, and the g-factor were examined in both cohorts. Full results are provided in **Supplementary Table 8a and Supplementary Figure 4.** Overall, CpG–MethylCog associations were large (|r| ≈ 0.30–0.75 across datasets). CpG–g associations were smaller but directionally consistent (|r| ≈ 0.05–0.32). Several CpGs showed highly reproducible associations across cohorts (e.g., cg10313673, cg03297163, cg17971015, cg21609339).

**Supplementary Table 8a.** HRS–HCAP Test Sample (n = 605): Pearson Correlations Between MethylCog, g, and CpG Sites

| **CpG Site** | ***r* (MethylCog)** | **95% CI** | ***p*** | ***r* (g)** | **95% CI** | ***p*** |
| --- | --- | --- | --- | --- | --- | --- |
| cg09190408 | –0.391 | –0.457, –0.321 | < .001 | –0.155 | –0.231, –0.076 | < .001 |
| cg10313673 | –0.555 | –0.608, –0.497 | < .001 | –0.181 | –0.257, –0.103 | < .001 |
| cg18181703 | 0.423 | 0.355, 0.486 | < .001 | 0.222 | 0.145, 0.297 | < .001 |
| cg26470501 | 0.377 | 0.307, 0.444 | < .001 | 0.190 | 0.112, 0.266 | < .001 |
| cg03297163 | –0.593 | –0.643, –0.539 | < .001 | –0.192 | –0.268, –0.114 | < .001 |
| cg04885881 | 0.538 | 0.479, 0.592 | < .001 | 0.217 | 0.140, 0.292 | < .001 |
| cg00687674 | –0.332 | –0.401, –0.259 | < .001 | –0.178 | –0.254, –0.100 | < .001 |
| cg18993757 | 0.037 | –0.043, 0.117 | .359 | 0.066 | –0.014, 0.145 | .105 |
| cg10504000 | 0.278 | 0.203, 0.350 | < .001 | 0.149 | 0.070, 0.226 | < .001 |
| cg17971015 | –0.516 | –0.573, –0.455 | < .001 | –0.152 | –0.229, –0.073 | < .001 |
| cg01657995 | 0.349 | 0.277, 0.417 | < .001 | 0.145 | 0.066, 0.223 | < .001 |
| cg21609339 | –0.601 | –0.650, –0.548 | < .001 | –0.210 | –0.285, –0.133 | < .001 |
| cg20859731 | –0.367 | –0.434, –0.296 | < .001 | –0.127 | –0.204, –0.047 | .002 |
| cg27152890 | –0.238 | –0.312, –0.162 | < .001 | –0.101 | –0.179, –0.021 | .013 |
| cg17222682 | 0.298 | 0.223, 0.369 | < .001 | 0.092 | 0.012, 0.171 | .024 |
| cg16736826 | 0.523 | 0.463, 0.579 | < .001 | 0.229 | 0.152, 0.303 | < .001 |
| cg21265568 | 0.012 | –0.068, 0.091 | .773 | 0.053 | –0.027, 0.132 | .196 |
| cg07537095 | –0.202 | –0.277, –0.124 | < .001 | –0.066 | –0.145, 0.014 | .106 |
| cg23023970 | 0.197 | 0.119, 0.272 | < .001 | 0.118 | 0.039, 0.196 | .004 |
| cg15705273 | –0.067 | –0.146, 0.013 | .098 | –0.025 | –0.105, 0.054 | .532 |
| cg18006990 | 0.080 | 0.000, 0.159 | .049 | 0.076 | –0.004, 0.155 | .063 |
| cg10636246 | 0.206 | 0.129, 0.281 | < .001 | 0.029 | –0.051, 0.109 | .477 |
| cg00947744 | 0.461 | 0.396, 0.521 | < .001 | 0.154 | 0.075, 0.231 | < .001 |
| cg01604883 | 0.407 | 0.338, 0.472 | < .001 | 0.151 | 0.072, 0.228 | < .001 |
| cg23988749 | –0.016 | –0.095, 0.064 | .699 | –0.008 | –0.088, 0.072 | .845 |
| cg05673882 | 0.333 | 0.260, 0.402 | < .001 | 0.177 | 0.098, 0.253 | < .001 |
| cg03636183 | 0.212 | 0.134, 0.287 | < .001 | 0.108 | 0.028, 0.186 | .008 |
| cg11828983 | –0.107 | –0.185, –0.027 | .009 | –0.036 | –0.116, 0.044 | .376 |
| cg03251079 | –0.544 | –0.598, –0.486 | < .001 | –0.210 | –0.285, –0.132 | < .001 |

Note. CI = confidence interval (95%).

**Supplementary Table 8b**. HBI Sample (n = 112): Pearson Correlations Between MethylCog, g, and CpG Sites

| **CpG Site** | ***r* (MethylCog)** | **95% CI** | ***p*** | ***r* (g)** | **95% CI** | ***p*** |
| --- | --- | --- | --- | --- | --- | --- |
| cg09190408 | –0.431 | –0.571, –0.267 | < .001 | –0.121 | –0.299, 0.067 | .206 |
| cg10313673 | –0.704 | –0.786, –0.596 | < .001 | –0.299 | –0.459, –0.121 | .001 |
| cg18181703 | 0.504 | 0.351, 0.630 | < .001 | 0.137 | –0.050, 0.314 | .151 |
| cg26470501 | 0.420 | 0.254, 0.561 | < .001 | 0.090 | –0.097, 0.271 | .345 |
| cg03297163 | –0.531 | –0.652, –0.383 | < .001 | –0.313 | –0.472, –0.136 | .001 |
| cg04885881 | 0.534 | 0.387, 0.655 | < .001 | 0.092 | –0.095, 0.273 | .333 |
| cg00687674 | –0.383 | –0.531, –0.213 | < .001 | –0.017 | –0.202, 0.169 | .856 |
| cg18993757 | 0.090 | –0.097, 0.271 | .345 | 0.104 | –0.083, 0.284 | .276 |
| cg10504000 | 0.289 | 0.109, 0.450 | .002 | 0.251 | 0.069, 0.417 | .008 |
| cg17971015 | –0.618 | –0.721, –0.489 | < .001 | –0.306 | –0.465, –0.128 | .001 |
| cg01657995 | 0.419 | 0.253, 0.561 | < .001 | 0.116 | –0.071, 0.296 | .221 |
| cg21609339 | –0.661 | –0.754, –0.542 | < .001 | –0.282 | –0.444, –0.102 | .003 |
| cg20859731 | –0.391 | –0.538, –0.222 | < .001 | –0.080 | –0.262, 0.107 | .399 |
| cg27152890 | –0.328 | –0.484, –0.151 | < .001 | –0.093 | –0.274, 0.094 | .329 |
| cg17222682 | 0.376 | 0.205, 0.525 | < .001 | 0.095 | –0.092, 0.276 | .318 |
| cg16736826 | 0.511 | 0.360, 0.636 | < .001 | 0.245 | 0.062, 0.411 | .009 |
| cg21265568 | –0.121 | –0.300, 0.066 | .204 | –0.023 | –0.207, 0.164 | .814 |
| cg07537095 | –0.157 | –0.333, 0.029 | .098 | –0.005 | –0.191, 0.181 | .957 |
| cg23023970 | 0.257 | 0.075, 0.422 | .006 | 0.157 | –0.030, 0.332 | .099 |
| cg15705273 | –0.403 | –0.548, –0.235 | < .001 | –0.039 | –0.223, 0.148 | .682 |
| cg18006990 | 0.064 | –0.124, 0.246 | .506 | 0.088 | –0.099, 0.269 | .356 |
| cg10636246 | 0.216 | 0.032, 0.386 | .022 | 0.110 | –0.077, 0.290 | .247 |
| cg00947744 | 0.546 | 0.402, 0.665 | < .001 | 0.132 | –0.055, 0.310 | .167 |
| cg01604883 | 0.508 | 0.356, 0.634 | < .001 | 0.172 | –0.014, 0.346 | .070 |
| cg23988749 | –0.088 | –0.269, 0.099 | .356 | –0.093 | –0.274, 0.094 | .329 |
| cg05673882 | 0.555 | 0.412, 0.671 | < .001 | 0.232 | 0.049, 0.400 | .014 |
| cg03636183 | 0.090 | –0.097, 0.271 | .345 | –0.096 | –0.276, 0.091 | .316 |
| cg11828983 | –0.182 | –0.356, 0.003 | .054 | –0.082 | –0.263, 0.105 | .392 |
| cg03251079 | –0.476 | –0.608, –0.318 | < .001 | –0.162 | –0.337, 0.025 | .088 |

**
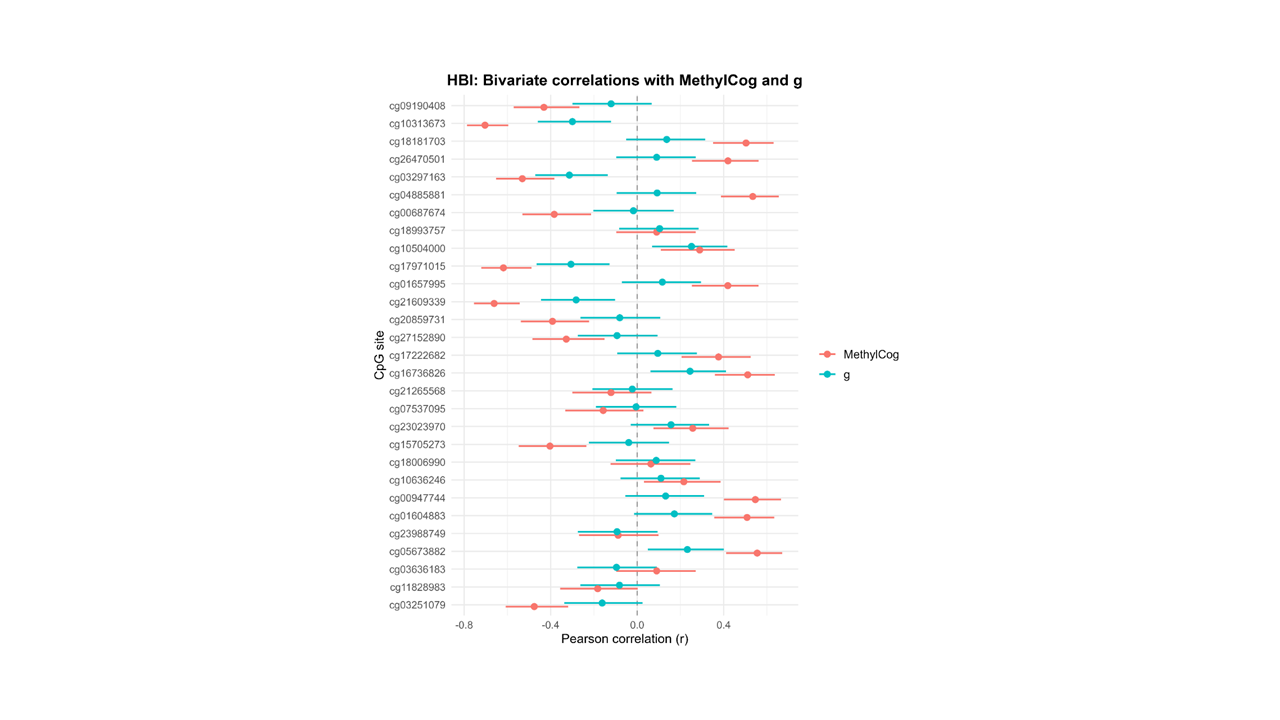
**

**Supplementary Figure 4.** Bivariate Pearson correlations between the 29 CpG sites and both MethylCog (red) and the general cognitive factor g (blue) in the HBI sample. Points represent correlation coefficients, and horizontal lines indicate 95% confidence intervals. Correlation directions were broadly consistent with those observed in HRS–HCAP but varied in magnitude due to smaller sample size.

**CpG Annotation and Genomic Context. Supplementary Table 9** contains full genomic annotation for all 29 CpGs including Genomic location (chromosome, position)**,** Gene name(s)**,** RefGene region (e.g., promoter, 5′UTR, body) and Relation to CpG island (e.g., island, shore, shelf, OpenSea). Given that the 29 CpGs span diverse biological processes, CpGs are grouped them functionally based on gene annotation and known pathways (**Supplementary Table 9**):

1. Neuroimmune and inflammatory signaling (e.g., SOCS3, AIM2, BCL3)
2. Neuronal/synaptic function and plasticity (e.g., CAMTA2, INPP5A, EDN2)
3. Epigenetic and chromatin remodeling genes (e.g., HMGA2, PPP1CA)
4. Cell adhesion and membrane signaling (e.g., CD81, C6orf48)
5. Transcriptional and regulatory genes

These groupings provide a biological framework for interpreting methylation signatures related to cognitive performance.

**Supplementary Table 9.** Genomic Annotation and Biological Grouping of the 29 CpG Sites Included in the MethylCog Model

A. Immune, Inflammatory, and Cytokine/Signaling Genes

| **CpG Site** | **Gene Symbol(s)** | **Full Gene Name(s)** | **chr** | **Position** | **Region** | **CpG Island Relation** |
| --- | --- | --- | --- | --- | --- | --- |
| **cg18181703** | SOCS3 | Suppressor of Cytokine Signaling 3 | chr17 | 76,354,621 | Body | N_Shore |
| **cg09190408** | PPP1CA / TBC1D10C | Protein Phosphatase 1 Catalytic Subunit A / TBC1 Domain Family Member 10C | chr11 | 67,170,610 | TSS1500 | S_Shore |
| **cg23988749** | MRPS18B / PPP1R10 | Mitochondrial Ribosomal Protein S18B / Protein Phosphatase 1 Regulatory Subunit 10 | chr6 | 30,585,293 | TSS200; TSS1500 | Island |
| **cg03636183** | F2RL3 | Coagulation Factor II Receptor-Like 3 (inflammatory signaling receptor) | chr19 | 17,000,585 | Body | N_Shore |
| **cg16736826** | EDN2 | Endothelin 2 (immune–vascular signaling) | chr1 | 41,951,512 | TSS1500 | OpenSea |
| **cg00947744** | NCAPH2 / LMF2 | Non-SMC Condensin II Complex Subunit H2 / Lipase Maturation Factor 2 | chr22 | 50,945,565 | TSS1500; Body | N_Shore |
| **cg23023970** | INPP5A | Inositol Polyphosphate-5-Phosphatase A (immune–metabolic signaling) | chr10 | 134,000,000 | Body | OpenSea |

B. Neural, Synaptic, and Calcium-Signaling Genes

| **CpG Site** | **Gene Symbol(s)** | **Full Gene Name(s)** | **chr** | **Position** | **Region** | **Relation** |
| --- | --- | --- | --- | --- | --- | --- |
| **cg18993757** | CAMTA2 | Calmodulin Binding Transcription Activator 2 | chr17 | 4,889,754 | 5′UTR; Body | N_Shore |
| **cg21609339** | SLC12A5 | Solute Carrier Family 12 Member 5 (KCC2 potassium-chloride cotransporter; neuron-specific) | chr20 | 44,650,270 | TSS200 | N_Shore |
| **cg04885881** | — | Intergenic near neuronal regulatory elements (non-coding) | chr1 | 11,123,118 | — | S_Shelf |
| **cg10313673** | CILP2 | Cartilage Intermediate Layer Protein 2 (neuronal ECM/intercellular signaling) | chr19 | 19,649,144 | 5′UTR; 1st Exon | Island |
| **cg05673882** | POLK | DNA Polymerase Kappa (neuronal DNA repair, damage tolerance) | chr5 | 74,862,702 | Body | OpenSea |

C. Epigenetic / Chromatin Structure and Transcription Regulation

| **CpG Site** | **Gene Symbol(s)** | **Full Gene Name(s)** | **chr** | **Position** | **Region** | **Relation** |
| --- | --- | --- | --- | --- | --- | --- |
| **cg26470501** | BCL3 | B-Cell Lymphoma 3 Protein (NF-κB transcriptional co-activator) | chr19 | 45,252,955 | Body | S_Shore |
| **cg03251079** | HMGA2 / RPSAP52 | High Mobility Group AT-Hook 2 (chromatin architecture) / RPSA Pseudogene 52 | chr12 | 66,218,285 | 1stExon; 5′UTR; Body | — |
| **cg10636246** | AIM2 | Absent in Melanoma 2 (inflammasome DNA-sensor) | chr1 | 159,000,000 | TSS1500 | OpenSea |
| **cg00687674** | MIR548H4 / TMEM84 | MicroRNA 548H4 (regulatory RNA) / Transmembrane Protein 84 | chr15 | 69,373,260 | Body | OpenSea |
| **cg01657995** | C6orf48 / SNORD52 | Chromosome 6 ORF48; Small Nucleolar RNA SNORD52 | chr6 | 31,804,883 | 5′UTR; Body | S_Shelf |

D. Membrane, Vesicle Transport, and Cell Adhesion Genes

| **CpG Site** | **Gene Symbol(s)** | **Full Gene Name(s)** | **chr** | **Position** | **Region** | **Relation** |
| --- | --- | --- | --- | --- | --- | --- |
| **cg10504000** | CD81 | CD81 Antigen (cell adhesion, synaptic modulation) | chr11 | 2,402,391 | Body | S_Shelf |
| **cg09190408** | PPP1CA / TBC1D10C | Actin cytoskeleton regulation / GTPase-activating protein | chr11 | 67,170,610 | TSS1500 | S_Shore |
| **cg00947744** | NCAPH2 / LMF2 | Nuclear condensin complex / lipid transport | chr22 | 50,945,565 | TSS1500; Body | N_Shore |

E. Genes Related to Metabolic, Structural, or Multi-Function Processes

| **CpG Site** | **Gene Symbol(s)** | **Full Gene Name(s)** | **chr** | **Position** | **Region** | **Relation** |
| --- | --- | --- | --- | --- | --- | --- |
| **cg27152890** | PPP1R13L | Protein Phosphatase 1 Regulatory Subunit 13L | chr19 | 45,900,241 | Body | Island |
| **cg21265568** | SPRYD3 | SPRY Domain Containing 3 | chr12 | 53,473,407 | TSS1500 | Island |
| **cg17971015** | — | Intergenic | chr19 | 12,306,198 | — | Island |
| **cg01604883** | — | Intergenic | chr5 | 150,000,000 | — | N_Shelf |
| **cg03636183** | F2RL3 | Coagulation Factor II Receptor-Like 3 | chr19 | 17,000,585 | Body | N_Shore |
| **cg07537095** | — | Intergenic | chr11 | 116,000,000 | — | OpenSea |
| **cg11828983** | — | Intergenic | chr14 | 105,000,000 | — | OpenSea |
| **cg17222682** | — | Intergenic | chr1 | 82,147,424 | — | OpenSea |
| **cg20859731** | — | Intergenic | chr20 | 36,226,820 | — | Island |

Note. Gene names are derived from UCSC RefGene and NCBI. Groupings reflect predominant biological functions associated with each gene.

**Sensitivity Analyses**

**APOE ε4 Status.** Sample characteristics stratified by APOE ε4 status are shown in **Supplementary Table S10**. In HRS Test, 23% (n=131) were ε4 carriers; in HBI, 29% (n=32) were carriers. MCI prevalence was higher among carriers in both cohorts (HRS: 33% vs. 20%; HBI: 34% vs. 33%).

**Supplementary Table 10**. Sample characteristics stratified by APOE ε4 status in the Test and HBI samples.

| Sample/Group | N | Age, M (SD) | Education, M (SD) | Female, % | MCI, n (%) | MethylCog, M (SD) | g, M (SD) |
| --- | --- | --- | --- | --- | --- | --- | --- |
| HRS Test Set |  |  |  |  |  |  |  |
| APOE ε4 non-carrier | 430 | 75.7 (7.4) | 12.8 (3.1) | 58.1 | 87 (20.2) | 0.08 (0.98) | 0.03 (0.99) |
| APOE ε4 carrier | 131 | 74.4 (6.0) | 12.9 (2.5) | 54.2 | 43 (32.8) | 0.19 (0.93) | -0.11 (1.04) |
|  |  |  |  |  |  |  |  |
| HBI |  |  |  |  |  |  |  |
| APOE ε4 non-carrier | 80 | 69.5 (10.4) | 16.1 (2.1) | 72.5 | 26 (32.5) | 2.66 (1.19) | 0.02 (0.97) |
| APOE ε4 carrier | 32 | 65.5 (10.2) | 16.4 (2.0) | 62.5 | 11 (34.4) | 2.37 (1.56) | -0.04 (1.09) |

**Main Effects Independent of APOE** MethylCog remained a significant predictor of general cognitive ability after adjusting for APOE ε4 carrier status in both cohorts (HRS Test: β = 0.196, p < .001; HBI: β = 0.216, p = .002). Similarly, MethylCog remained significantly associated with MCI status after APOE adjustment in HRS Test (OR = 0.70, p = .002) and showed a trend in HBI (OR = 0.68, p = .033). MethylCog coefficients were unchanged when APOE was added to models, indicating independence from genetic risk.

**Effect Modification** MethylCog showed numerically larger effect sizes in APOE ε4 carriers compared to non-carriers (**Supplementary Table 11)**. In HRS Test, carriers showed more than double the incremental variance explained (ΔR² = 5.4% vs. 2.4%) and stronger protective effects against MCI (OR = 0.54 vs. 0.76). A similar pattern emerged in HBI (carriers: ΔR² = 19.8%, OR = 0.50; non-carriers: ΔR² = 2.6%, OR = 0.81), though small sample sizes limited precision (n = 32 carriers). The MethylCog × APOE interaction reached trend-level significance for continuous cognition in HRS Test (β = 0.156, p = .046) but not in HBI (β = 0.172, p = .215) or for MCI outcomes (ps ≥ .092). Given the modest subgroup sizes, these findings require replication before drawing conclusions about differential validity by genetic risk status.

**Supplementary Table 11.** Effect Modification by APOE ε4 Carrier Status on MethylCog Performance

| Sample/Group | N | r [95% CI]ᵃ | p | β [95% CI]ᵇ | p | ΔR² | N (MCI) | OR [95% CI]ᶜ | p | AUC (Base → Full) |
| --- | --- | --- | --- | --- | --- | --- | --- | --- | --- | --- |
| HRS Test Set |  |  |  |  |  |  |  |  |  |  |
| Non-carrier | 430 | 0.41 [0.33, 0.48] | <.001 | 0.17 [0.10, 0.25] | <.001 | 2.40% | 87 | 0.76 [0.58, 0.98] | 0.03 | 0.601 → 0.628 (Δ0.027) |
| Carrier | 131 | 0.48 [0.33, 0.60] | <.001 | 0.28 [0.13, 0.43] | <.001 | 5.40% | 43 | 0.54 [0.34, 0.87] | 0.01 | 0.666 → 0.724 (Δ0.057) |
| Interaction test |  |  |  | β = 0.156, p = .046 | |  |  | OR = 0.65, p = .092 | | |
|  |  |  |  |  |  |  |  |  |  |  |
| HBI |  |  |  |  |  |  |  |  |  |  |
| Non-carrier | 80 | 0.29 [0.08, 0.48] | 0.008 | 0.14 [−0.03, 0.31] | 0.11 | 2.60% | 26 | 0.81 [0.50, 1.32] | 0.4 | 0.754 → 0.764 (Δ0.010) |
| Carrier | 32 | 0.52 [0.21, 0.73] | 0.002 | 0.38 [0.11, 0.64] | 0.01 | 19.80% | 11 | 0.50 [0.23, 1.09] | 0.08 | 0.706 → 0.861 (Δ0.156) |
| Interaction test |  |  |  | β = 0.172, p = .215 | |  |  | OR = 0.68, p = .353 | | |

Note. All linear regression models predicting general cognitive ability (g) adjusted for age, sex, and education. All logistic regression models predicting mild cognitive impairment (MCI) vs. cognitively unimpaired status adjusted for age, sex, and education. CI = confidence interval; OR = odds ratio; AUC = area under the receiver operating characteristic curve.

ᵃ Pearson correlation between MethylCog and general cognitive ability (g).

ᵇ Standardized regression coefficient for MethylCog in fully adjusted linear model.

ᶜ Odds ratio for MethylCog in fully adjusted logistic model (OR < 1 indicates lower odds of MCI).

**Sensitivity Analysis: Race/Ethnicity** Both cohorts had limited racial/ethnic diversity (HRS Test: 85% White; HBI: 86% White). Among Non-White participants in HRS Test (n=92), 82% identified as Black/African American. The HBI Non-White group (n=16) was too small for granular subgroup analyses

**Main Effects Independent of Race** MethylCog remained a significant predictor after adjusting for race/ethnicity in both cohorts (HRS Test: β = 0.115, p = .004; HBI: β = 0.216, p = .033). In models without education adjustment, MethylCog showed comparable effect sizes across racial/ethnic groups (HRS Test White: β = 0.266, p < .001; Non-White: β = 0.207, p = .066). When education was added as a covariate, a trend-level interaction emerged in HRS Test (p = .060) but not in HBI (p = .937). However, formal three-way interaction testing (MethylCog × race × education) was non-significant in both cohorts (ps ≥ .843), indicating education did not meaningfully moderate race-specific effects. These findings suggest MethylCog's associations do not differ substantially by race/ethnicity, though limited sample sizes in Non-White subgroups preclude definitive conclusions.

**Mediation Analyses in relation to race/ethnicity and education**

**Education as Mediator of MethylCog → Cognition**

First we examined whether education (as an early life course exposure and documented driver of race/ethnic disparities in cognitive aging) significantly mediated the association between MethylCog and general cognitive ability in HRS Test. Results showed that education accounted for a substantial proportion of the MethylCog-g association in the test set (43.0% mediation effect; ACME = 0.151, 95% CI [0.114, 0.192], p < .001), with trend-level mediation in HBI (14.0% mediated; ACME = 0.036, 95% CI [-0.001, 0.084], p = .060). Both the MethylCog → education path (path a) and the education → cognition path (path b) were significant, indicating that education statistically accounted for part of the association between MethylCog and cognition. However, the direct effect of MethylCog on cognition remained highly significant in both cohorts (both ps ≤ .002), demonstrating MethylCog predicts cognition through both educational and non-educational pathways.

**MethylCog as Mediator of Race → Cognition**

MethylCog significantly mediated racial/ethnic differences in g in the test and HBI sets (**Supplementary Table 11**). In models adjusting for age and sex only, Non-White participants showed significantly lower MethylCog scores (HRS Test: β = -1.12, p < .001; HBI: β = -1.78, p < .001), and these differences statistically accounted for substantial proportions of observed cognitive disparities (HRS: 32.9% mediated, ACME = -0.29, 95% CI [-0.39, -0.20], p < .001; HBI: 45.1% mediated, ACME = -0.36, 95% CI [-0.73, -0.04], p = .020). Mediation persisted after adjusting for educational attainment. In HRS Test, 14.3% of racial disparities remained mediated by MethylCog independent of education (ACME = -0.10, 95% CI [-0.17, -0.04], p < .001), with the race → MethylCog pathway minimally affected by education adjustment (β = -1.04, p < .001). In HBI, a similar pattern emerged with 39.4% mediated after education adjustment (ACME = -0.28, 95% CI [-0.61, 0.02], p = .066), retaining 80% of the unadjusted mediation effect. These findings suggests that

MethylCog may reflect cognition-relevant biological correlates of social and environmental exposures that are unevenly distributed across racialized groups and (2) are not fully accounted for by formal educational attainment. For MCI outcomes, the total effect of race was non-significant in HRS Test (OR = 1.47, p = .150) but became essentially null after accounting for MethylCog (OR = 1.03, p = .931), s. In HBI, the race effect on MCI was stronger (OR = 3.70, p = .030) and was substantially attenuated though not eliminated after accounting for MethylCog (OR = 2.22, p = .282). However, these MCI analyses should be interpreted cautiously given limited numbers of Non-White participants with MCI (HRS Test n=24; HBI n=8). Together, these exploratory analyses suggest that MethylCog may reflect cumulative life-course exposures relevant to cognitive aging, some of which are partially indexed by education but not fully explained by it.

**Supplementary Table 12.** Direct and Indirect effects with MethylCog as a mediator of the race-g association (age and sex adjusted)

| **Dataset** | **N** | **Total effect (c)** | **p** | **Direct effect (c′)** | **p** | **Indirect effect (ab)** | **p** | **Proportion mediated** |
| --- | --- | --- | --- | --- | --- | --- | --- | --- |
| HRS Test | 605 | −0.88 | < .001 | −0.59 | < .001 | −0.29 | < .001 | .33 |
| HBI | 112 | −0.79 | .003 | −0.44 | .132 | −0.36 | .020 | .45 |

**Differences in MethylCog by Demographic and Clinical Characteristics** Results are summarized in **Supplementary Table 12.** In the HBI set, MethylCog scores differed significantly by diagnostic status (**Supplementary Figure 5**), with cognitively normal individuals (M = 2.92, SD = 1.19) scoring significantly higher than those with MCI (M = 2.08, SD = 1.34), t(69.5) = 3.13, p = .003. A significant difference was also observed across racial groups **(Supplementary Figure 6),** F(1.0, 16.7) = 10.88, p = .004, with White participants (M = 2.84, SD = 1.24) scoring higher than participants from other racial/ethnic minority groups (M = 1.34, SD = 1.33).

Age was negatively correlated with MethylCog scores, r(110) = -.23, p = .01, indicating lower scores with increasing age **(Supplementary Figure 7a)**. To further examine age effects, participants were categorized into three groups: younger than 65 years (n = 39, M = 2.95, SD = 1.36), 65-75 years (n = 38, M = 2.62, SD = 1.13), and 76 years and older (n = 35, M = 2.12, SD = 1.33). Welch's ANOVA revealed a significant difference across these age groups **(Supplementary Figure 7b)**, F(2, 71.6) = 3.49, p = .036, with MethylCog scores declining across successive age categories.

Years of education showed a positive but non-significant correlation with MethylCog scores, r(110) = .15, p = .11 (S**upplementary Figure 8a)** .When education was categorized into two groups (11-16 years: n = 93, M = 2.57, SD = 1.30 vs. 17+ years: n = 19, M = 2.80, SD = 1.27), the difference remained non-significant, t(110) = -0.77, p = .443 **(Supplementary Figure 8b)**.

No significant differences in MethylCog scores were observed for sex, t(61.9) = -0.10, p = .92 **(Supplementary Figure 9)**., APOE ε4 carrier status **(Supplementary Figure 10)**., t(46.1) = 0.94, p = .35, or Hispanic ethnicity, t(11.8) = -0.16, p = .88 **(Supplementary Figure 11)**.

In the **HRS test** set, MethylCog scores differed significantly by diagnostic status **(Supplementary Figure 5)**., with cognitively normal individuals (M = 0.14, SD = 0.96) scoring significantly higher than those with mild cognitive impairment (M = -0.48, SD = 0.92), t(242.2) = 4.11, p < .001.

Significant differences were also observed across race groups **(Supplementary Figure 6)**, F(2, 38.7) = 46.09, p < .001, with higher MethylCog scores in the White group (M = 0.34, SD = 0.86) compared to Black/African American group (M = -0.64, SD = 0.91) and the Other race category (M = -0.61, SD = 1.04). Age was negatively correlated with MethylCog scores, r(603) = -.20, p < .001 (**Supplementary Figure 7a**). When participants were divided into two age groups (65-75 years: n = 325, M = 0.20, SD = 1.03 vs. 76+ years: n = 280, M = -0.02, SD = 0.87), the younger group scored significantly higher than the older group, t(603) = 2.81, p = .005 (**Supplementary Figure 7b**). Years of education were positively correlated with MethylCog scores, r(603) = .34, p < .001(**Supplementary Figure 7a**. This relationship was further supported by categorical analysis across three education groups: 0-10 years (n = 88, M = -0.59, SD = 0.87), 11-16 years (n = 437, M = 0.14, SD = 0.95), and 17+ years (n = 80, M = 0.64, SD = 0.76), F(2, 159) = 48.2, p < .001 **(Supplementary Figure 7)**. Hispanic participants (M = -0.53, SD = 1.01) scored significantly lower than non-Hispanic participants (M = 0.14, SD = 0.95), t(77.0) = 5.19, p < .001 (**Supplementary Figure 10**) No significant differences were observed for sex, t(582.2) = -0.18, p = .85 **(Supplementary Figure 8**) or APOE ε4 carrier status, t(224.5) = -1.17, p = .24 **(Supplementary Figure 8)**.

**
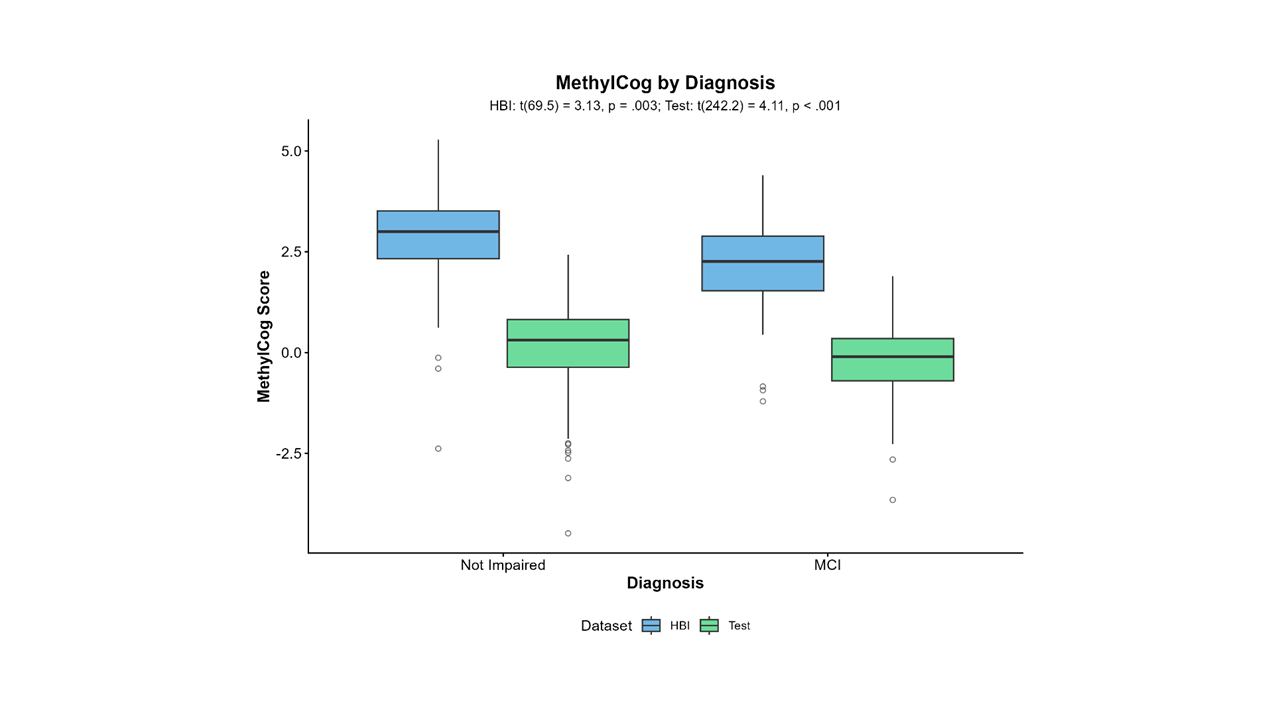
**

**Supplementary Figure 5** Box plots showing MethylCog scores by diagnostic status (cognitively normal vs. mild cognitive impairment) in the HBI and HRS test datasets. Both datasets show significantly lower MethylCog scores in individuals with MCI compared to cognitively normal individuals.


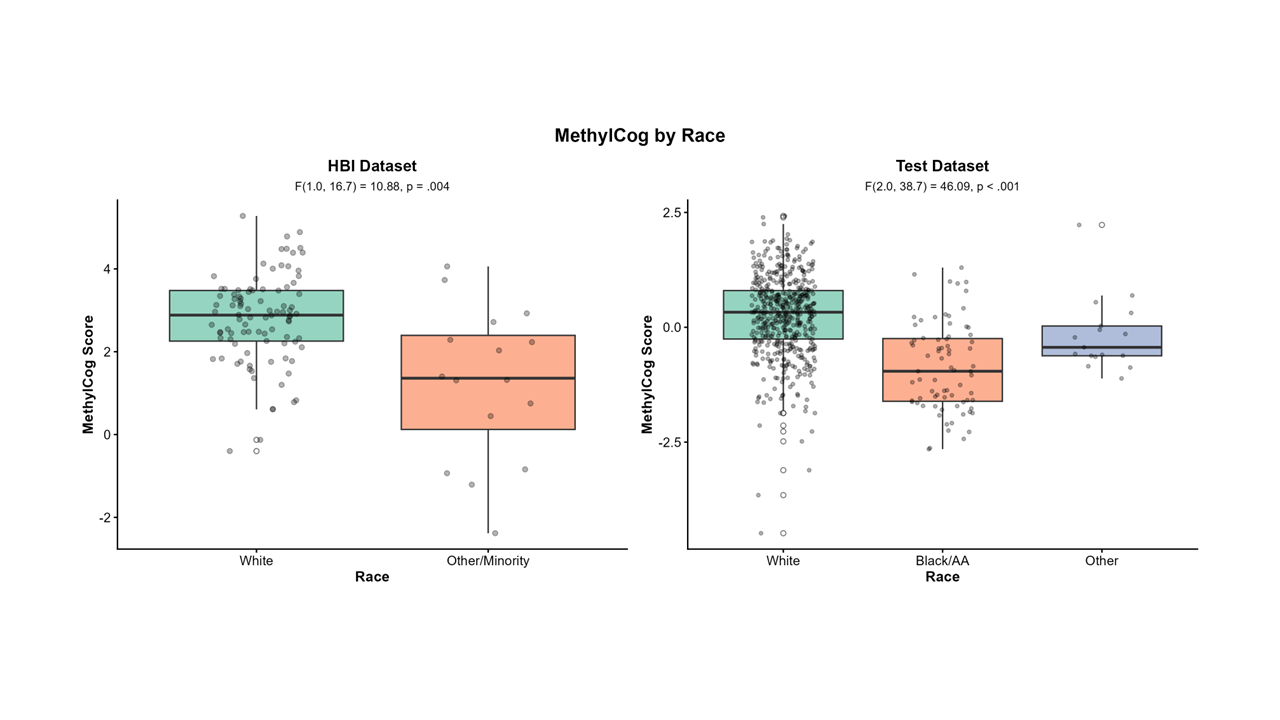


**Supplementary Figure 6** Box plots showing MethylCog scores by race in the HBI dataset (White vs. Other/Minority) and HRS test dataset (White vs. Black/African American vs. Other). Both datasets show significant racial disparities in MethylCog scores.


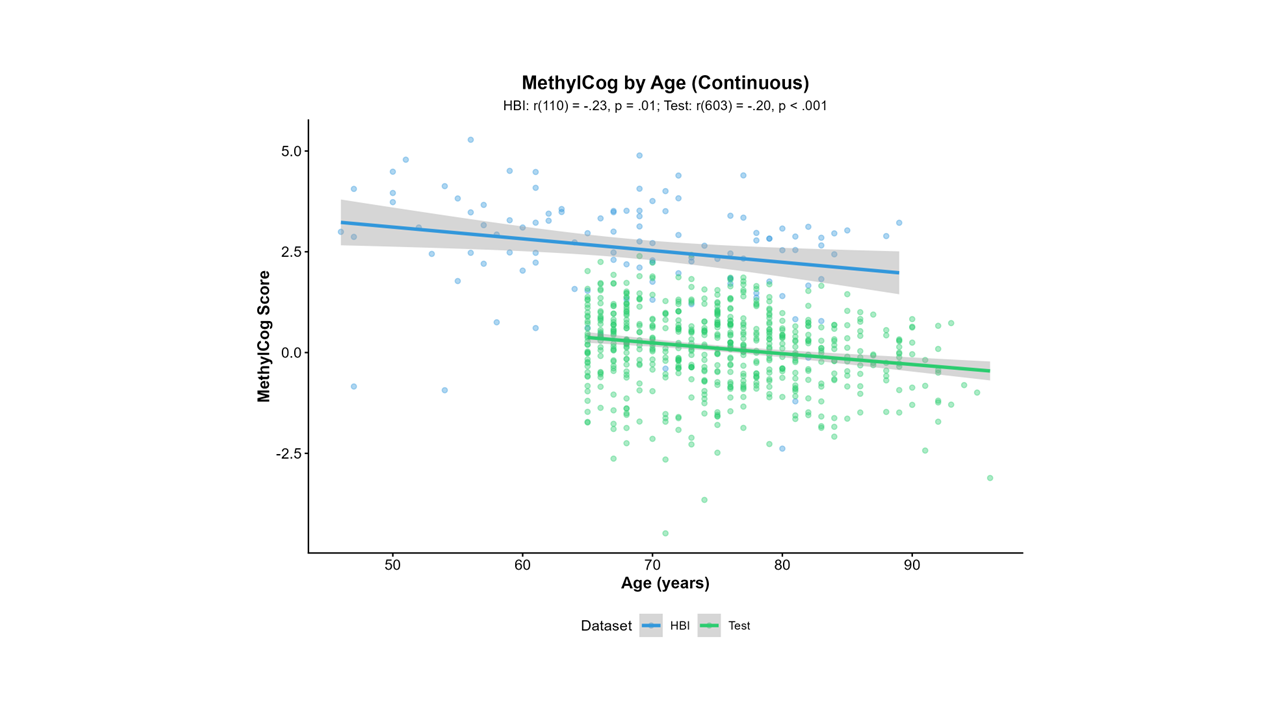


**Supplementary Figure 7a** Scatter plots showing the relationship between age (continuous) and MethylCog scores in the HBI and HRS test datasets. Both datasets show significant negative correlations, with regression lines and 95% confidence intervals displayed.


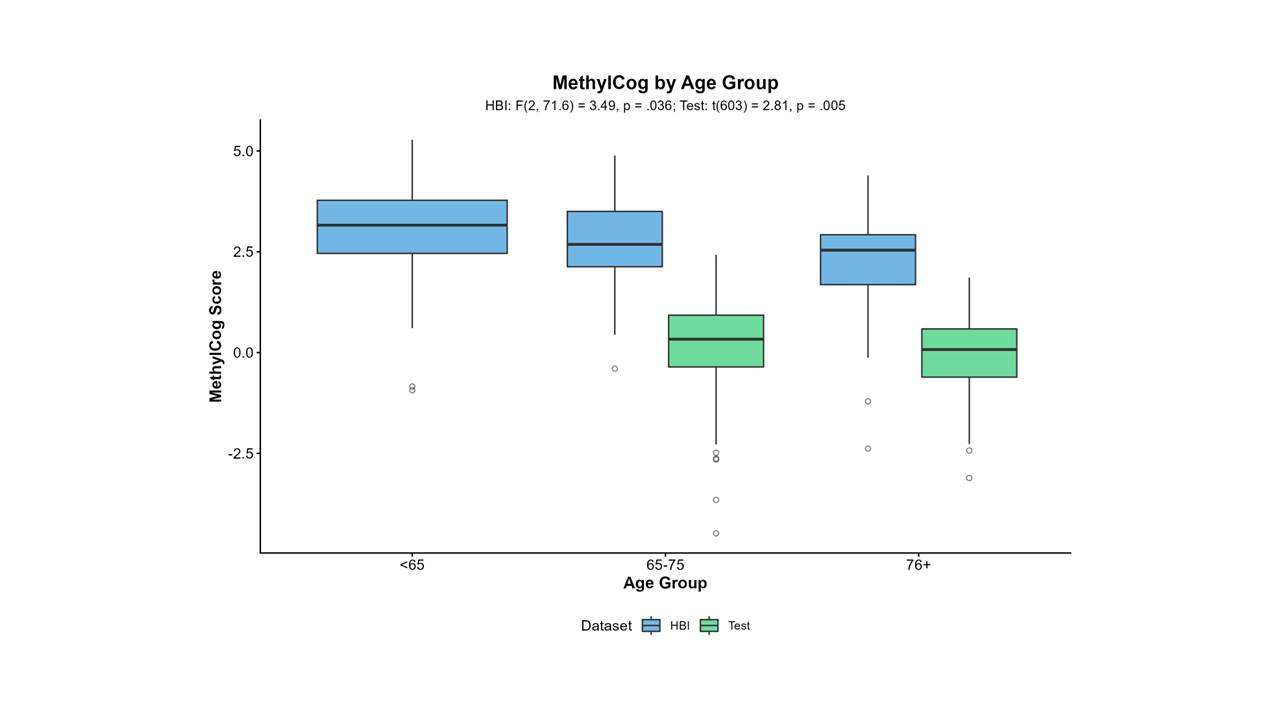


**Supplementary Figure 7b** Box plots showing MethylCog scores by age group in the HBI dataset (<65, 65-75, 76+ years) and HRS test dataset (65-75, 76+ years). Both datasets demonstrate age-related differences in MethylCog scores.


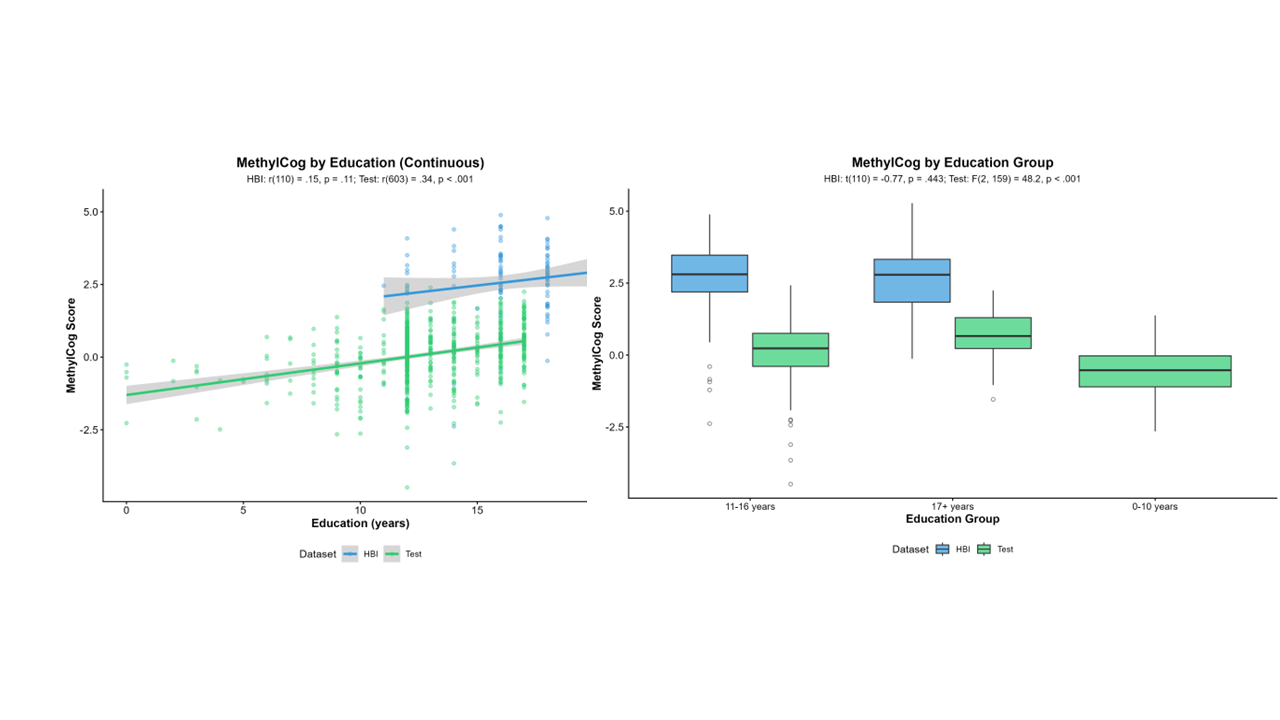


(B)

(A)

S**upplementary Figure 8A and 8B** (A) Scatterplot showing the association between years of education and MethylCog scores.
(B) Group comparison of MethylCog scores by educational attainment category. No statistically significant differences were observed across education groups.


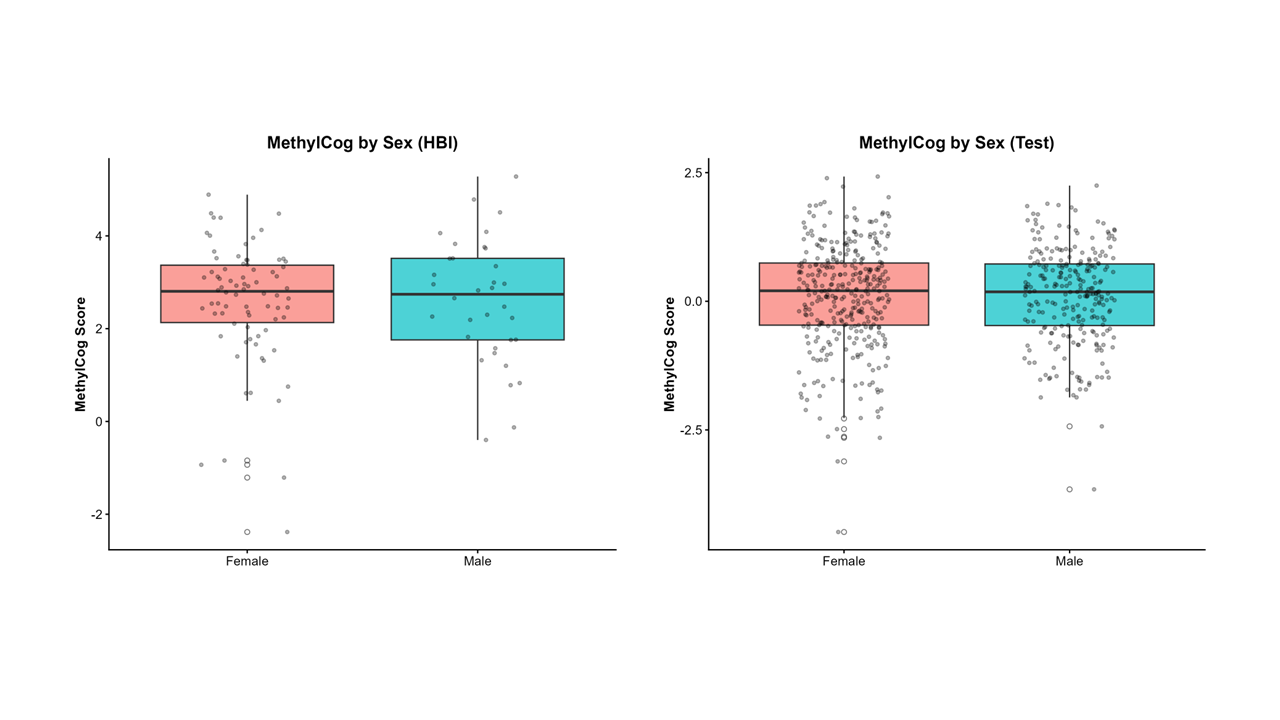


**Supplementary Figure 9** Distribution of MethylCog scores by sex. No significant differences were observed between males and females.


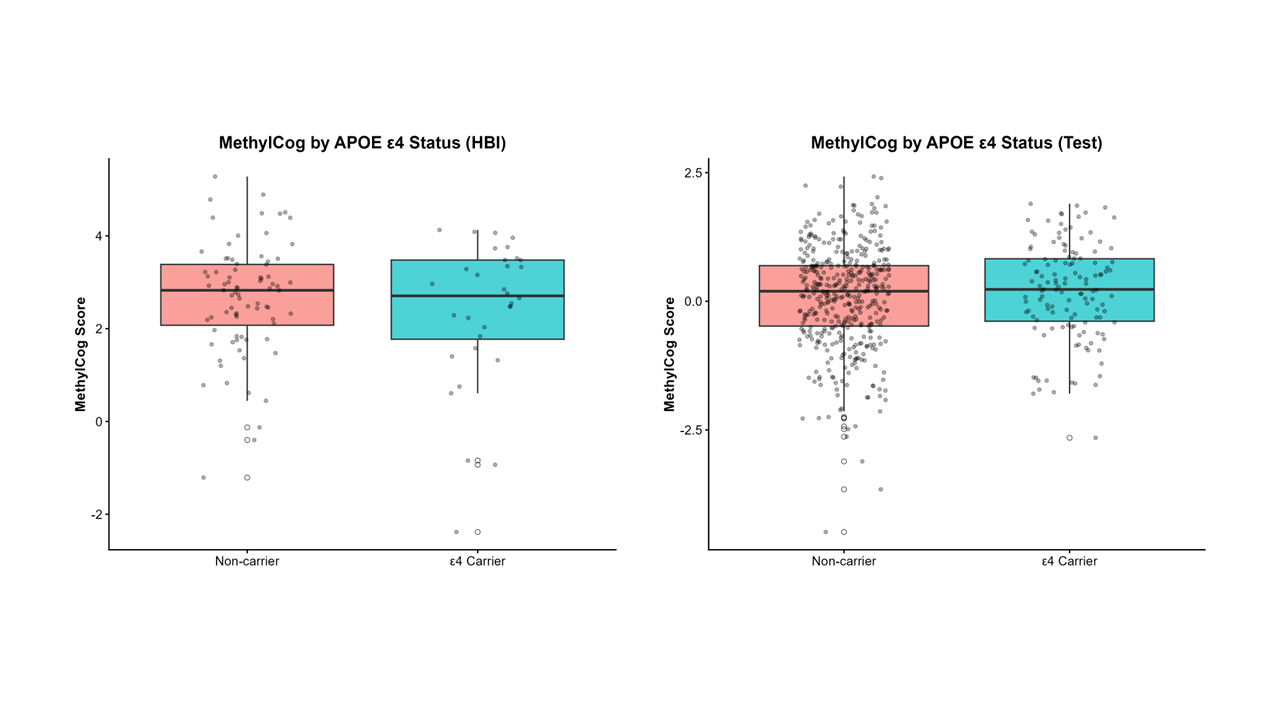


**Supplementary Figure 10** Distribution of MethylCog scores by APOE status. No significant differences were observed between e4 carriers and non-carriers


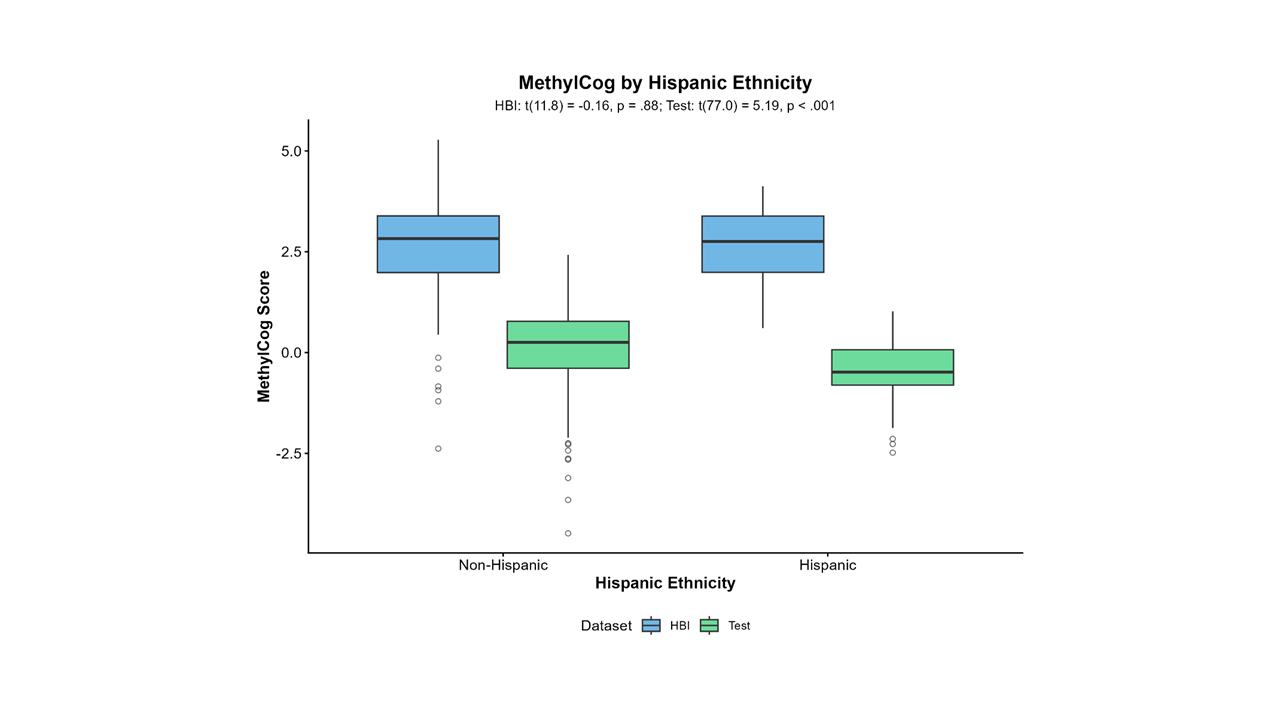


**Supplementary Figure 11** Box plots showing MethylCog scores by Hispanic ethnicity (non-Hispanic vs. Hispanic) in the HBI and HRS test datasets. Significant differences were observed in the test dataset only.

**Supplementary** **Table 13.** Descriptive Statistics and Group Comparisons for MethylCog Scores in HBI and HRS Test Datasets

| **Variable** | **Dataset** | **Group** | ***n*** | ***M*** | ***SD*** | **Test Statistic** | ***p*** |
| --- | --- | --- | --- | --- | --- | --- | --- |
| **Sex** |  |  |  |  |  |  |  |
|  | HBI | Female | 56 | 2.63 | 1.31 | *t*(61.9) = -0.10 | .92 |
|  |  | Male | 56 | 2.66 | 1.27 |  |  |
|  | Test | Female | 338 | 0.10 | 0.97 | *t*(582.2) = -0.18 | .85 |
|  |  | Male | 267 | 0.12 | 0.96 |  |  |
| **Diagnosis** |  |  |  |  |  |  |  |
|  | HBI | Not Impaired | 63 | 2.92 | 1.19 | *t*(69.5) = 3.13 | .003 |
|  |  | MCI | 49 | 2.08 | 1.34 |  |  |
|  | Test | Not Impaired | 431 | 0.14 | 0.96 | *t*(242.2) = 4.11 | <.001 |
|  |  | MCI | 174 | -0.48 | 0.92 |  |  |
| **APOE ε4** |  |  |  |  |  |  |  |
|  | HBI | Non-carrier | 75 | 2.56 | 1.29 | *t*(46.1) = 0.94 | .35 |
|  |  | Carrier | 37 | 2.26 | 1.28 |  |  |
|  | Test | Non-carrier | 375 | 0.15 | 0.96 | *t*(224.5) = -1.17 | .24 |
|  |  | Carrier | 230 | 0.05 | 0.97 |  |  |
| **Hispanic Ethnicity** |  |  |  |  |  |  |  |
|  | HBI | Non-Hispanic | 101 | 2.65 | 1.30 | *t*(11.8) = -0.16 | .88 |
|  |  | Hispanic | 11 | 2.73 | 1.22 |  |  |
|  | Test | Non-Hispanic | 531 | 0.14 | 0.95 | *t*(77.0) = 5.19 | <.001 |
|  |  | Hispanic | 74 | -0.53 | 1.01 |  |  |
| **Race** |  |  |  |  |  |  |  |
|  | HBI | White | 97 | 2.84 | 1.24 | *F*(1.0, 16.7) = 10.88 | .004 |
|  |  | Other/Minority | 15 | 1.34 | 1.33 |  |  |
|  | Test | White | 496 | 0.34 | 0.86 | *F*(2.0, 38.7) = 46.09 | <.001 |
|  |  | Black/AA | 71 | -0.64 | 0.91 |  |  |
|  |  | Other | 38 | -0.61 | 1.04 |  |  |
| **Age Group** |  |  |  |  |  |  |  |
|  | HBI | <65 years | 39 | 2.95 | 1.36 | *F*(2, 71.6) = 3.49 | .036 |
|  |  | 65-75 years | 38 | 2.62 | 1.13 |  |  |
|  |  | 76+ years | 35 | 2.12 | 1.33 |  |  |
|  | Test | 65-75 years | 325 | 0.20 | 1.03 | *t*(603) = 2.81 | .005 |
|  |  | 76+ years | 280 | -0.02 | 0.87 |  |  |
| **Education Group** |  |  |  |  |  |  |  |
|  | HBI | 11-16 years | 93 | 2.57 | 1.30 | *t*(110) = -0.77 | .443 |
|  |  | 17+ years | 19 | 2.80 | 1.27 |  |  |
|  | Test | 0-10 years | 88 | -0.59 | 0.87 | *F*(2, 159) = 48.2 | <.001 |
|  |  | 11-16 years | 437 | 0.14 | 0.95 |  |  |
|  |  | 17+ years | 80 | 0.64 | 0.76 |  |  |
| **Age (continuous)** |  |  |  |  |  |  |  |
|  | HBI | — | 112 | 68.0 | 10.5 | *r* = -.23 | .01 |
|  | Test | — | 605 | 75.5 | 7.0 | *r* = -.20 | <.001 |
| **Education (continuous)** |  |  |  |  |  |  |  |
|  | HBI | — | 112 | 15.4 | 2.1 | *r* = .15 | .11 |
|  | Test | — | 605 | 13.1 | 2.7 | *r* = .34 | <.001 |

*Note.* HBI = Healthy Brain Initiative; Test = Health and Retirement Study test set; MCI = mild cognitive impairment; APOE ε4 = apolipoprotein E ε4 allele; AA = African American. Welch's *t*-tests were used for binary comparisons, Welch's ANOVA for categorical variables with three or more levels, and Pearson's product-moment correlations for continuous variables. Statistically significant results (*p* < .05) are shown in bold. Sample sizes may not sum to total due to missing data for some variables.


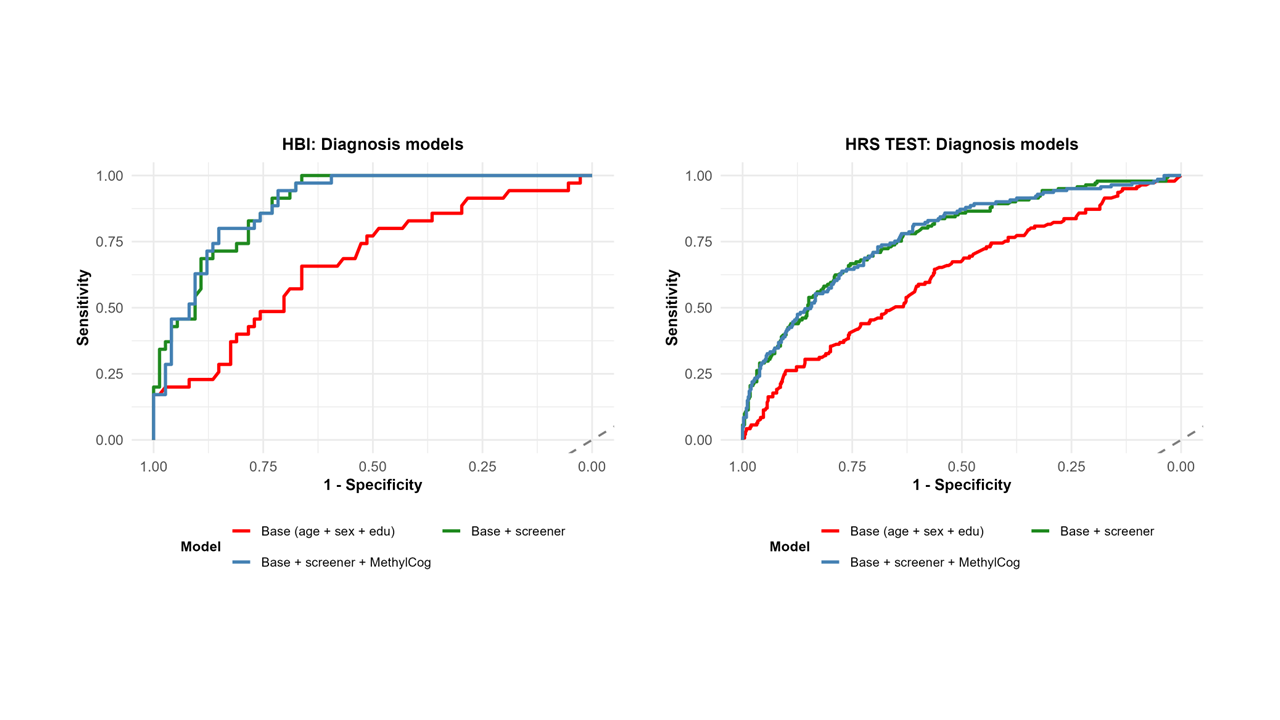


**Supplementary Figure 12** Receiver operating characteristic (ROC) curves illustrate discrimination of cognitively unimpaired versus MCI status using models including demographics and a cognitive screener (MMSE in the HRS test set; MoCA in the HBI sample), with and without MethylCog. Cognitive screeners demonstrated good discrimination (AUCs = 0.77–0.89), and inclusion of MethylCog resulted in negligible changes in AUC (ΔAUCs ≤ 0.001) and no independent association with MCI diagnosis.

**Bivariate Correlations among GrimAge, MethylCog, general cognitive ability and age**

Bivariate correlations among MethylCog, cognitive ability, chronological age, and epigenetic age (GrimAge) were examined in both samples using Pearson's product-moment correlations.

**HBI Set Results** In the HBI set, MethylCog was significantly positively correlated with g_fac, *r*(110) = .38, *p* < .001, 95% CI [.21, .53], and significantly negatively correlated with both chronological age, *r*(110) = -.23, *p* = .014, 95% CI [-.40, -.05], and GrimAge, *r*(110) = -.32, *p* < .001, 95% CI [-.48, -.14]. Cognitive ability (g) was negatively associated with both chronological age, *r*(110) = -.25, *p* = .008, 95% CI [-.42, -.07], and GrimAge, *r*(110) = -.27, *p* = .003, 95% CI [-.44, -.09]. As expected, chronological age and GrimAge were very strongly correlated, *r*(110) = .91, *p* < .001, 95% CI [.87, .94] **(Supplementary Figure 13)**.

**HRS Test Set Results** in the HRS test set closely replicated the HBI findings. MethylCog was significantly positively correlated with g_fac, *r*(603) = .41, *p* < .001, 95% CI [.34, .47], and significantly negatively correlated with chronological age, *r*(603) = -.20, *p* < .001, 95% CI [-.27, -.12], and GrimAge, *r*(603) = -.35, *p* < .001, 95% CI [-.42, -.28]. Cognitive ability (g_fac) showed stronger negative associations with both chronological age, *r*(603) = -.43, *p* < .001, 95% CI [-.50, -.37], and GrimAge, *r*(603) = -.47, *p* < .001, 95% CI [-.53, -.41], compared to the HBI set. Chronological age and GrimAge were again strongly correlated, *r*(603) = .77, *p* < .001, 95% CI [.74, .80](**Supplementary Figure 13)**.


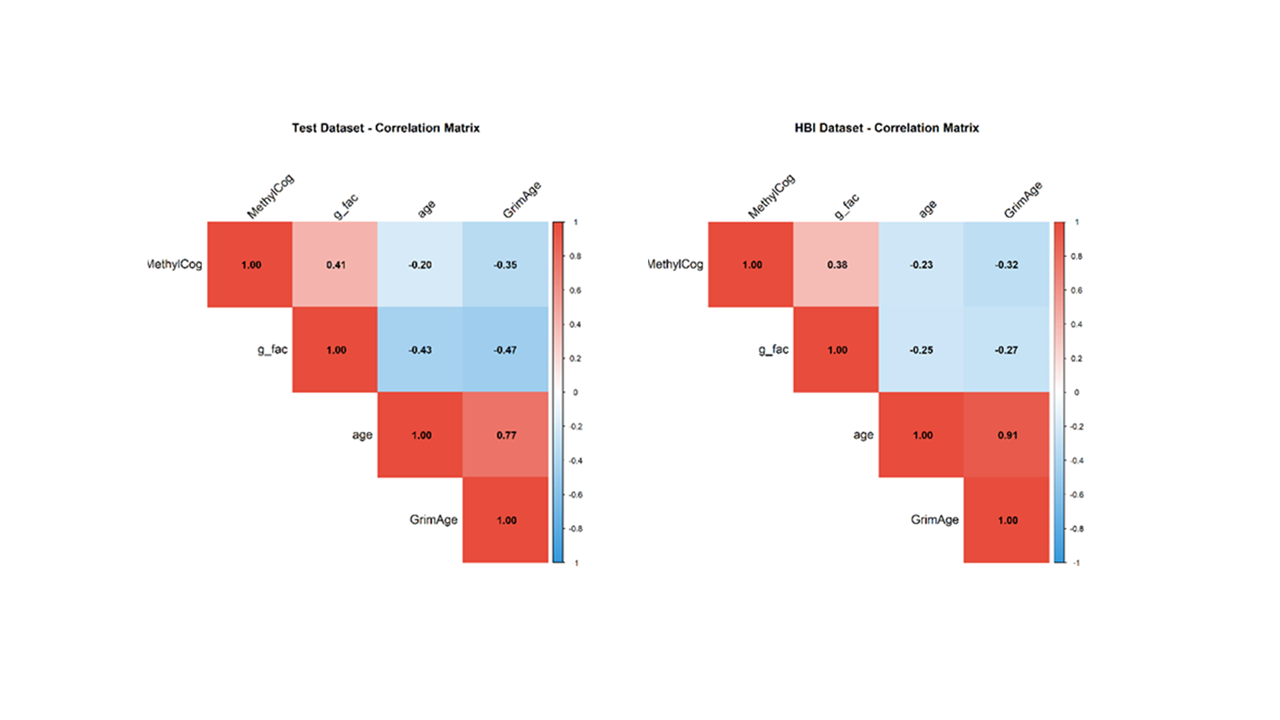


**Supplementary Figure 13**  Bivariate correlation matrices for MethylCog, general cognitive ability (g_fac), chronological age, and GrimAge in the HRS test (n = 605) and HBI (n = 112) datasets. Red indicates positive correlations; blue indicates negative correlations. MethylCog showed moderate positive associations with g_fac and negative associations with age and GrimAge in both samples. All correlations p < .05.

**Partial Correlations Controlling for Age** To examine whether the observed associations between MethylCog, cognitive ability, and epigenetic age were independent of chronological age, partial correlations controlling for age were computed for both samples.

**HBI Set Results** After controlling for chronological age, the association between MethylCog and g_fac remained significant and moderate in strength, *r*(109) = .32, *p* < .001, 95% CI [.14, .48], representing a modest attenuation from the zero-order correlation. This indicates that MethylCog captures variance in cognitive ability beyond age-related effects.

The negative association between MethylCog and GrimAge also remained significant after age adjustment, *r*(109) = -.24, *p* = .011, 95% CI [-.41, -.06], though attenuated from the zero-order correlation. This suggests that MethylCog is associated with accelerated epigenetic aging independent of chronological age. Notably, the correlation between g_fac and GrimAge became non-significant after controlling for age, *r*(109) = -.03, *p* = .752, 95% CI [-.22, .16], indicating that this relationship was primarily driven by shared age variance (**Supplementary Figure 14)**.

**HRS Test Set Results** in the HRS test set closely replicated the HBI findings. The association between MethylCog and g_fac remained robust after controlling for age, *r*(602) = .32, *p* < .001, 95% CI [.24, .39], representing an attenuation from the zero-order correlation. This replicates the finding that MethylCog captures cognitive ability independent of age.

Similarly, the negative association between MethylCog and GrimAge remained significant after age adjustment, *r*(602) = -.25, *p* < .001, 95% CI [-.33, -.18], attenuated from the zero-order correlation, again supporting the interpretation that MethylCog is associated with accelerated biological aging beyond chronological age effects. The correlation between g_fac and GrimAge, while significantly attenuated, remained significant after controlling for age, *r*(602) = -.14, *p* < .001, 95% CI [-.22, -.06], compared to the substantial zero-order correlation (**Supplementary Figure 14)**.

**Summary** Across both samples, MethylCog demonstrated significant positive associations with cognitive ability and negative associations with both chronological and epigenetic age. Partial correlations revealed that MethylCog's associations with cognitive ability (g_fac) and epigenetic aging (GrimAge) persist after controlling for chronological age, though with expected attenuation. The MethylCog-g_fac partial correlation showed consistency across samples (*r* = .32 in both HBI and test sets), demonstrating that approximately 10% of the variance in cognitive ability is captured by MethylCog independent of age. The persistent negative associations with GrimAge after age adjustment suggest that MethylCog may capture aspects of accelerated biological aging that are relevant to cognitive function.These findings provide strong evidence that MethylCog is not merely a proxy for chronological age but represents meaningful biological variation related to cognitive performance and aging processes.


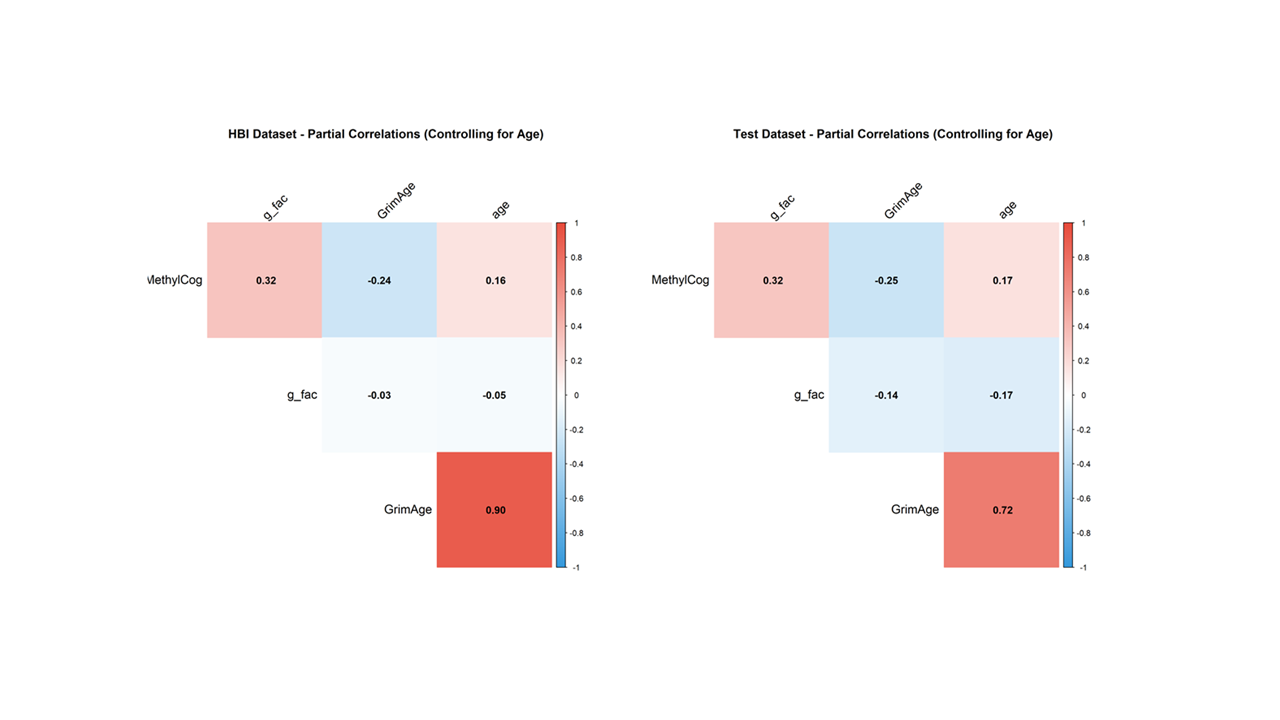


**Supplementary Figure 14.** Partial correlations among MethylCog, general cognitive ability (g_fac), and GrimAge controlling for chronological age in HBI (n = 112) and HRS test (n = 605) datasets. Red indicates positive correlations; blue indicates negative correlations. After age adjustment, MethylCog remained significantly associated with g_fac (r = .32 in both samples) and GrimAge (both r ≈ -.25), whereas g_fac-GrimAge associations were substantially weakened. Results demonstrate MethylCog's age-independent associations with cognitive ability and epigenetic aging.

**Supplementary Table 14** Zero-Order and Partial Correlations Among MethylCog, Cognitive Ability, Age, and Epigenetic Age

| **Dataset** | **Variable Pair** | ***r*** | ***p*** | **95% CI** | ***R partial* ~^a^** | ***p*** | **95% CI** |
| --- | --- | --- | --- | --- | --- | --- | --- |
| **HBI (*n* = 112)** |  |  |  |  |  |  |  |
|  | MethylCog — g_fac | .38*** | <.001 | [.21, .53] | .32*** | <.001 | [.14, .48] |
|  | MethylCog — Age | -.23* | .014 | [-.40, -.05] | — | — | — |
|  | MethylCog — GrimAge | -.32*** | <.001 | [-.48, -.14] | -.24* | .011 | [-.41, -.06] |
|  | g_fac — Age | -.25** | .008 | [-.42, -.07] | — | — | — |
|  | g_fac — GrimAge | -.27** | .003 | [-.44, -.09] | -.03 | .752 | [-.22, .16] |
|  | Age — GrimAge | .91*** | <.001 | [.87, .94] | — | — | — |
| **Test (*n* = 605)** |  |  |  |  |  |  |  |
|  | MethylCog — g_fac | .41*** | <.001 | [.34, .47] | .32*** | <.001 | [.24, .39] |
|  | MethylCog — Age | -.20*** | <.001 | [-.27, -.12] | — | — | — |
|  | MethylCog — GrimAge | -.35*** | <.001 | [-.42, -.28] | -.25*** | <.001 | [-.33, -.18] |
|  | g_fac — Age | -.43*** | <.001 | [-.50, -.37] | — | — | — |
|  | g_fac — GrimAge | -.47*** | <.001 | [-.53, -.41] | -.14*** | <.001 | [-.22, -.06] |
|  | Age — GrimAge | .77*** | <.001 | [.74, .80] | — | — | — |

*Note.* CI = confidence interval; g_fac = general cognitive ability factor; GrimAge = DNA methylation-based estimate of phenotypic age.^a^ Partial correlations control for chronological age.

* *p* < .05. ** *p* < .01. *** *p* < .001.

**Associations with ADRD biomarkers and MRI (HBI only)**


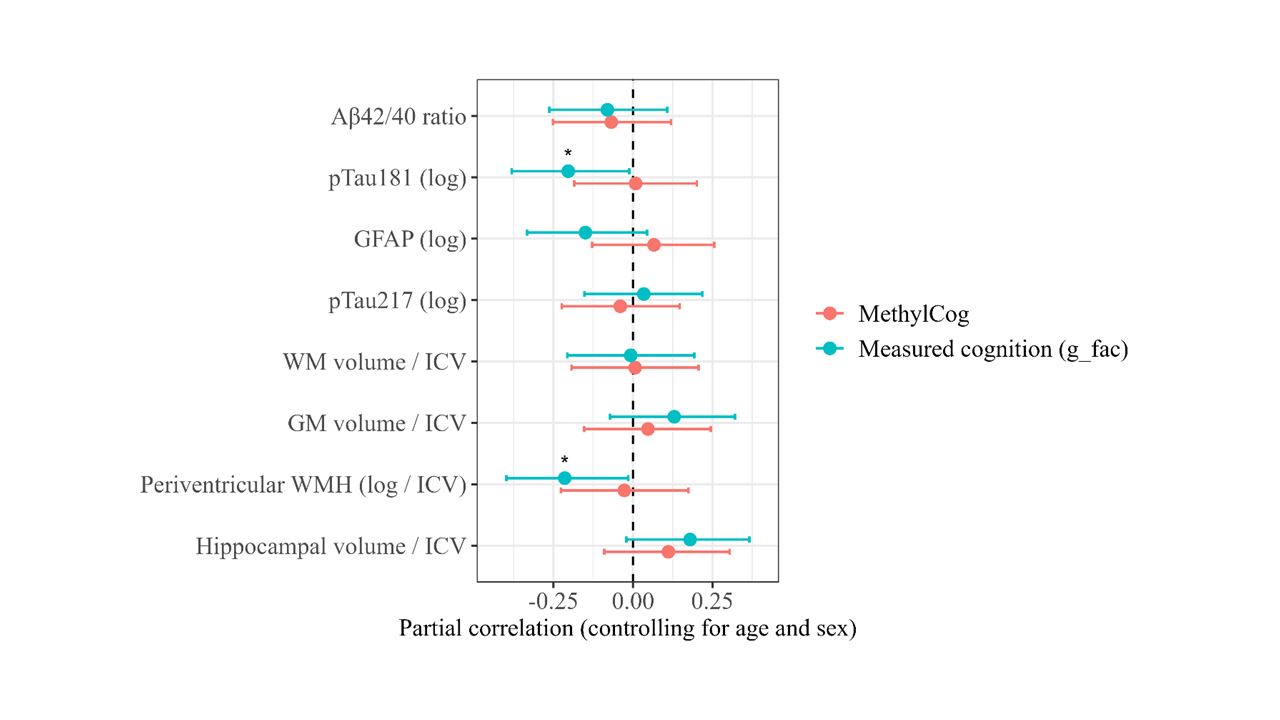


**Supplementary Figure 15** Partial correlations between MethylCog, g, plasma ADRD biomarkers, and MRI measures.


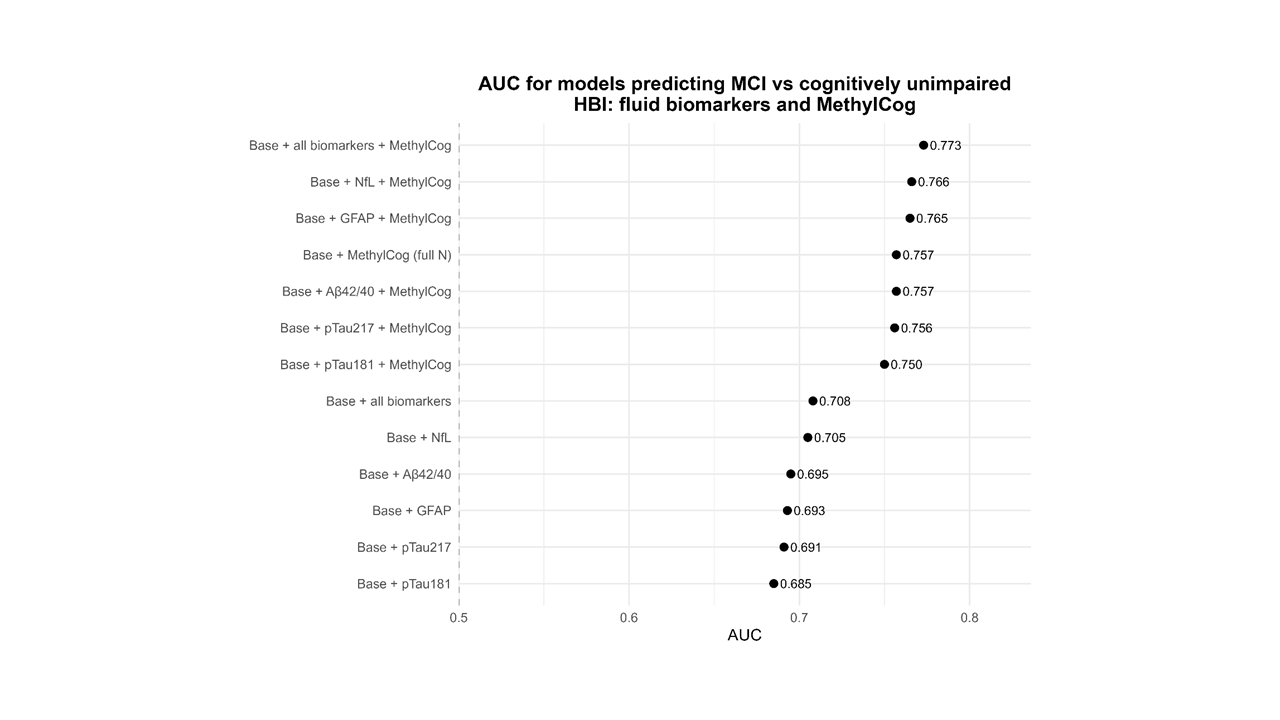


### **Supplementary Figure 16. Discrimination of MCI using fluid biomarker and MethylCog models.** Dot plots display area under the ROC curve (AUC) values for models including demographics alone, demographics plus individual fluid biomarkers, MethylCog alone, and combined biomarker + MethylCog models. Models that included MethylCog achieved AUCs of approximately 0.75–0.77, exceeding biomarker-only models and closely matching MethylCog-only performance, indicating that MethylCog captured most of the discriminative signal provided by fluid biomarkers.

**
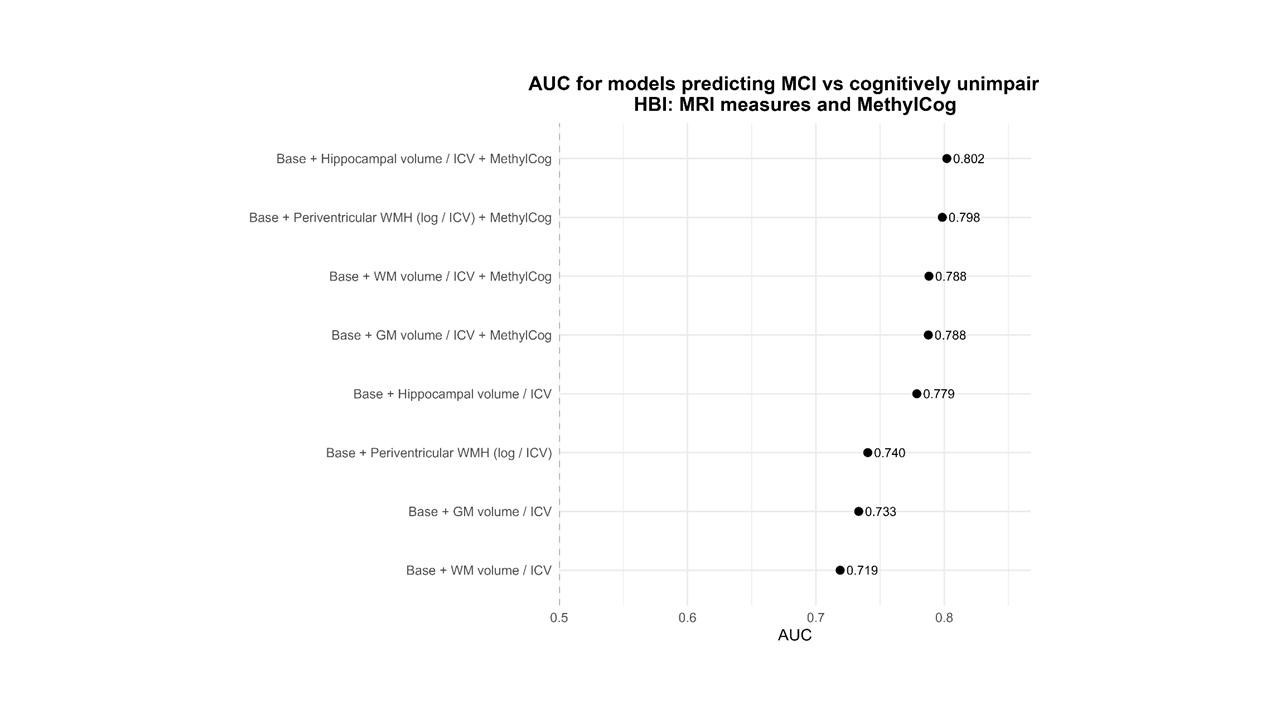
**

### **Supplementary Figure 17. Incremental discrimination gained by adding MethylCog to MRI-based models.** Dot plots show AUCs for MRI-based models including demographics plus gray matter volume, hippocampal volume, or periventricular white matter hyperintensity (WMH), with and without MethylCog. The largest improvement was observed when MethylCog was added to the periventricular WMH model, increasing AUC from approximately 0.74 to 0.80, with comparable gains observed for gray matter and hippocampal volume models.

**References**

1. SCHERR, P.A., et al., *CORRELATES OF COGNITIVE FUNCTION IN AN ELDERLY COMMUNITY POPULATION.* American Journal of Epidemiology, 1988. **128**(5): p. 1084-1101.

2. Wechsler, D., *Wechsler memory scale-revised.* Psychological Corporation, 1987.

3. Heyman, A., et al., *The Consortium to Establish a Registry for Alzheimer's Disease (CERAD). Part I. Clinical and neuropsychological assesment of Alzheimer's disease.* Neurology, 1989. **39**(9): p. 1159-1159.

4. Lachman, M.E., et al., *Monitoring cognitive functioning: psychometric properties of the brief test of adult cognition by telephone.* Assessment, 2014. **21**(4): p. 404-417.

5. Smith, A., *Symbol digit modalities test.* Los Angeles, CA, 2013.

6. Steptoe, A., et al., *Cohort profile: the English longitudinal study of ageing.* International journal of epidemiology, 2013. **42**(6): p. 1640-1648.

7. Armitage, S.G., *An analysis of certain psychological tests used for the evaluation of brain injury.* Psychological monographs, 1946. **60**(1): p. i.

8. Wilson, R.S., et al., *Participation in cognitively stimulating activities and risk of incident Alzheimer disease.* Jama, 2002. **287**(6): p. 742-748.

9. Fisher, G., et al., *New measures of fluid intelligence in the HRS*. 2014.

10. Woodcock, R.W., K.S. McGrew, and N. Mather, *Woodcock-Johnson III tests of achievement.* 2001.

11. Folstein, M., S. Folstein, and P. McHugh, *5.2 Mini-mental state examination (MMSE).* Manual of screeners for dementia, 2020. **51**.

12. Nasreddine, Z.S., et al., *The Montreal Cognitive Assessment, MoCA: a brief screening tool for mild cognitive impairment.* Journal of the American Geriatrics Society, 2005. **53**(4): p. 695-699.

13. Benedict, R.H., et al., *Hopkins Verbal Learning Test–Revised: Normative data and analysis of inter-form and test-retest reliability.* The Clinical Neuropsychologist, 1998. **12**(1): p. 43-55.

14. Bruno, D., et al., *Story recall performance and AT classification via positron emission tomography: A comparison of logical memory and Craft Story 21.* Journal of the Neurological Sciences, 2024. **464**: p. 123148.

15. Bahri, M., et al., *Brain Gray Matter Volumes and Age-Related Changes in Visual Episodic Memory: Insights from the Benson Complex Figure Test.* Available at SSRN 5517029.

16. Sattler, J.M. and J.J. Ryan, *Assessment with the WAIS-IV*. 2009: Jerome M Sattler Publisher.

17. Galvin, J.E., et al., *The Number Symbol Coding Task: A brief measure of executive function to detect dementia and cognitive impairment.* PLoS One, 2020. **15**(11): p. e0242233.

18. Stasenko, A., et al., *The Multilingual Naming Test (MINT) as a measure of picture naming ability in Alzheimer’s disease.* Journal of the International Neuropsychological Society, 2019. **25**(8): p. 821-833.
